# Radioembolization of Hepatocellular Carcinoma with ^90^Y Glass Microspheres: No Advantage of Voxel Dosimetry with Respect to Mean Dose in Dose–Response Analysis with Two Radiological Methods

**DOI:** 10.3390/cancers14040959

**Published:** 2022-02-15

**Authors:** Chiara Romanò, Stefania Mazzaglia, Marco Maccauro, Carlo Spreafico, Alejandro Gabutti, Gabriele Maffi, Carlo Morosi, Tommaso Cascella, Marta Mira, Maria Chiara De Nile, Gianluca Aliberti, Giovanni Argiroffi, Valentina Fuoco, Sherrie Bhoori, Consuelo Zanette, Alfonso Marchianò, Ettore Seregni, Vincenzo Mazzaferro, Carlo Chiesa

**Affiliations:** 1 Nuclear Medicine, Foundation IRCCS Istituto Nazionale Tumori, 20133 Milan, Italy; chiara.romano@asst-santipaolocarlo.it (C.R.); stefania.mazzaglia@istitutotumori.mi.it (S.M.); marco.maccauro@istitutotumori.mi.it (M.M.); marta.mira@asst-ovestmi.it (M.M.); gianluca.aliberti@istitutotumori.mi.it (G.A.); giovanni.argiroffi@istitutotumori.mi.it (G.A.); valentina.fuoco@istitutotumori.mi.it (V.F.); consuelo.zanette@istitutotumori.mi.it (C.Z.); ettore.seregni@istitutotumori.mi.it (E.S.); 2 Radiology Foundation IRCCS Istituto Nazionale Tumori, 20133 Milan, Italy; carlo.spreafico@istitutotumori.mi.it (C.S.); jesus.gabuttit@incmnsz.mx (A.G.); gabriele.maffi@apss.tn.it (G.M.); carlo.morosi@istitutotumori.mi.it (C.M.); tommaso.cascella@istitutotumori.mi.it (T.C.); alfonso.marchiano@istitutotumori.mi.it (A.M.); 3 Postgraduate Specialization School in Medical Physics, University of Milan, 20133 Milan, Italy; mcdenile@hotmail.it; 4 HPB Surgery, Hepatology and Liver Transplantation, Foundation IRCCS Istituto Nazionale Tumori, 20133 Milan, Italy; sherrie.bhoori@istitutotumori.mi.it (S.B.); vincenzo.mazzaferro@istitutotumori.mi.it (V.M.)

**Keywords:** TARE, SIRT, liver radioembolization, treatment planning, dosimetry, response assessment, voxel, radiobiological models

## Abstract

**Simple Summary:**

We confirmed that the non-uniformity of an intra-lesion dose distribution, which was introduced in calculations as voxel dosimetry, did not significantly improve the AUC values of the dose–response relationship with respect to the mean dose. This was probably derived from the strong correlations (all *p* < 0.0001) among all voxel-based dosimetric variables (minimum Spearman correlation coefficient: 0.67) caused by the limited spatial resolution of nuclear medicine images. Responses were assessed with mRECIST and with an experimental densitometric method with a response threshold optimized at 20% HU variation. Significant dose–response agreement was obtained only with the densitometric method and only with post-therapy 90Y-PET data. More unexpectedly, the injection of Theraspheres™ on day 8 from the reference date rather than on day 4 worsened the dose–response correlation and reduced the efficacy at high doses. This may be explained by the increased non-uniformity following the non-linear mega-clustering effect triggered by the higher number of microspheres/GBq injected on day 8.

**Abstract:**

In this confirmatory study, we tested if a calculation that included the non-uniformity of dose deposition through a voxel-based dosimetric variable *Ψ* was able to improve the dose–response agreement with respect to the mean absorbed dose D. We performed dosimetry with ^99m^Tc-MAA SPECT/CT and ^90^Y-PET/CT in 86 patients treated 8 instead of 4 days after the reference date with 2.8 times more ^90^Y glass microspheres/GBq than in our previous study. The lesion-by-lesion response was assessed with the mRECIST method and with an experimental densitometric criterion. A total of 106 lesions were studied. Considering *Ψ* as a prognostic response marker, having no *Ψ* provided a significantly higher AUC than D. The correlation, *t*-test, and AUC values were statistically significant only with the densitometric method and only with post-therapy dosimetry. In comparison with our previous study, the dose–response correlation and AUC values were poorer (maximum r = 0.43, R^2^ = 0.14, maximal AUC = 0.71), and the efficacy at a high dose did not reach 100%. The expected advantages of voxel dosimetry were nullified by the correlation between any *Ψ* and D due to the limited image spatial resolution. The lower AUC and efficacy may be explained by the mega-clustering effect triggered by the higher number of microspheres/GBq injected on day 8.

## 1. Introduction

Trans-arterial radioembolization (TARE) is a locoregional therapy for both primary and metastatic liver malignancies, and it is performed by injecting radioactive microspheres [1,2]. Despite the initial promising results obtained in phase II studies [3,4,5], it failed to demonstrate its superiority over sorafenib in two prospective randomized phase III studies on locally advanced hepatocellular carcinoma (HCC) patients (SIRveNIB [6], SARAH [7]), as well as in three phase III studies on colorectal metastases (FOXFIRE, SIRFLOX, and FOXFIRE-Global) [8]. Among the possible reasons for such failures, the lack of optimization of the therapy through personalized absorbed-dose calculation (dosimetry) was proposed [9]. Indeed, the search for the optimal TARE outcome through individualized dosimetry has been a field of research for our group since the beginning of TARE [10]. Differently from systemic radiopharmaceuticals, dosimetric calculations for microspheres are performed by exploiting a single SPECT/CT or PET/CT scan, which is ordinarily performed for clinical reasons. Given the simplicity of this procedure and the potential clinical implications, dosimetry is nowadays a main research stream for TARE [11]. Additional motivations derive from the striking success of the DOSISPHERE-01 study [12]. This prospective randomized phase II study showed a median overall survival gain in locally advanced HCC from 10.7 to 26.6 months using tumor/non-tumor dosimetry versus standard dosage. This huge gain demonstrates the impact of dosimetry on clinical outcomes. The Dosimetry Committee of the European Association of Nuclear Medicine (EANM) recently published guidelines for ^90^Y microsphere dosimetry [13].

In greater detail, a few weeks before radioembolization, ^99m^Tc albumin-macroaggregated (^99m^Tc-MAA) is injected intra-arterially to evaluate possible lung shunt and to exclude the deposition of gastro-enteric microspheres, which is an absolute contraindication for treatment. The ^99m^Tc-MAA SPECT/CT images obtained allow pre-treatment dosimetry or, in other words, treatment simulation and planning, as in external beam radiotherapy (EBRT). The glass microspheres used for therapy in this study were loaded with ^90^Y, an almost pure beta- emitter, which also has an extremely low probability of beta+ emission (3.186 ± 0.047) × 10^−5^ [14]. This allows ^90^Y-positron emission tomography (PET) imaging and post-therapy dosimetry [15].

Basic dosimetry evaluates the mean absorbed dose in the volumes of interest (VOIs) in tumors, non-tumoral tissue, and the lungs with simple calculation methods [13]. This approach neglects the potential role of the non-uniformity of absorbed-dose deposition in tissues. The most sophisticated, advanced, and intriguing method for including the heterogeneity of microsphere distribution in dosimetric calculations evaluates the absorbed dose at the voxel level (voxel = VOlumetric piXEL), i.e., at the smallest accessible scale of a tomographic image, including radiobiology. Dose volume histograms (DVHs) are obtained, as in EBRT. However, the actual advantages of voxel dosimetry in nuclear medicine therapy are under debate and have not yet been demonstrated [16]. For this reason, the EANM guidelines indicate that the mean dose approach is mandatory, while the voxel calculation is optional [13]. The main aim of the present work was to test the improvement of the dosimetric prediction of clinical outcome through all possible voxel-based calculations.

In our previous study aiming at such a demonstration [17], contrarily to our expectations, the radiobiological voxel dosimetry provided only a negligible advantage with respect to the mean dose. However, recent papers seem to confirm the validity of the voxel approach in TARE dosimetry [18,19]. We therefore performed a critical analysis of our previous study [17], and the following flaws were identified.

- The radiological response criterion was experimental and did not have a consensus for its application to hepatocellular carcinoma (HCC) response after TARE. It was an extreme variation of the densitometric method by Choi et al. [20]. The threshold for radiological response along the follow-up arterial-phase CT scans was arbitrarily fixed at a 50% reduction of tumor Hounsfield units (HUs), while in the original work [20], it was at 15%.
- Image scatter correction license was not available. This could have caused, on average, a overestimation of the absorbed-dose value in non-tumoral livers by 15%, with a range from −20% to +35% [21].
- A hybrid SPECT-CT scanner was not available. Volume delineation was performed on pure SPECT images.
- Peri-therapy with ^90^Y PET verification was not performed.
- The number of studied lesions was limited to 60.
- The number of dosimetric variables *Ψ* considered was limited to 4, and only radiobiological parameters were calculated.

The main aim of the present study was to repeat the search for the best predictor/descriptor of clinical outcomes with the inclusion of the non-uniformity of microsphere distribution in the calculations once the above-mentioned flaws were solved (primary endpoint). A larger number of voxel dosimetry variables *Ψ* were considered, as well as a larger number of lesions.

As a secondary endpoint, we aimed to confirm or renew the dose–toxicity (normal tissue complication probability, NTCP) and dose–response (tumor control probability, TCP) relationships obtained in [17]. Since these also depend on the response assessment method, we investigated the accuracies of the available radiological response evaluation methods. Gavanier et al. [22] demonstrated that the densitometric method proposed by Choi et al. is more suitable for accessing the response of HCC to systemic sorafenib than mRECIST, the method that has been consolidated for TARE [23]. During our previous SPETc-DOSE-1 study (approved by the Ethics Committee with the number INT 99-17 and accomplished in 2017–2018), we searched for the best threshold of the method of Choi et al. to apply to TARE of HCC. The best threshold value shifted from 15% to 20%. This research is described in the Supplementary Material, which should be read as a preliminary pre-requisite, not as optional information, for the present paper. We remark that the present study does not aim to validate the densitometric method. It only reports interesting results that could stimulate researchers to undertake clinical validation studies.

The present study is rather complex and covers several aspects. To help the reader, we summarize the logical structure in the following:

(1) Primary endpoint: determining which dosimetric voxel-based variables *Ψ* provide the best agreement with radiological response; no voxel-based variables improved the agreement of the dose–response relationship with respect to the mean dose (a confirmed failure of voxel dosimetry despite the methodological advances).
(2) Secondary endpoint: comparing the dose–response relationship obtained with two radiological response assessment methods (mRECIST and the densitometric method with a 20% HU reduction threshold). Surprisingly, the experimental densitometric method outperformed mRECIST, as it was the only one that gave a significant absorbed-dose difference between responding and non-responding lesions.
(3) Secondary endpoint: pursuing the primary endpoint using pre- and post-treatment dosimetric data and comparing the results obtained; only post-therapy dosimetry gave a significant absorbed-dose difference between responding and non-responding lesions.
(4) Important unexpected observations deserving future focused study: indication of the worsening of the dose–response correlation, of the dose–response agreement, and of the reduction of the efficacy injecting glass microspheres 8 versus 4 days after the reference date, i.e., using an increased number of microspheres/GBq with respect to [17].

The present work is the first part of the ongoing NumberDose study for TARE, which has been approved by the Ethics Committee of our institution (INT 154-19).

## 2. Materials and Methods

The methodology of the present work was conceived to solve all of the methodological limitations of our previous study [17]:

- Use of a proper radiological response assessment method;
- Advanced hybrid ^99m^Tc-MAA SPECT/CT images (with scatter correction and quantitative reconstruction available);
- Systematic post-therapy ^90^Y PET/CT dosimetric verification;
- A larger number of patients (175) and lesions (106);
- A larger number of voxel-based dosimetric parameters, including non-radiobiological parameters.

### 2.1. Inclusion Criteria and Treatment

Our inclusion criteria for intermediate/advanced hepatocarcinoma (HCC) patients and our methodology for TARE are described in [24]. We considered a series of patients treated with ^90^Y glass microspheres (THERASPHERE™ produced by Boston Scientific, Marlborough, MA, USA) beginning on 14 September 2015, and we started treating patients on Monday of the second week, 7.75 days after the calibration date, until the end of 2018. Microspheres were always injected into a single lobe in each therapy session. If necessary, a second treatment was performed after an interval of at least of 6 months to accomplish the radio-induced hypertrophy. Toxicity and efficacy data from after the second treatment were not included in the analysis. Super-selective administration was used for 22% of the patients considered.

In order to have a cohort similar to that of our previous study, especially regarding liver tolerance to radiation, the following additional inclusion criteria were retrospectively applied:

Well-compensated cirrhosis (Child-Pugh A) [25];
Tumor burden < 50%;
No previous TARE or concomitant sorafenib;
Portal vein thrombosis (PVT) grade < IIIb [26] (exclusion of cases with complete obstruction of the main trunk).

Treatment was planned with ^99m^Tc-MAA SPECT/CT images with a multi-compartment dosimetric approach (lung, lesions, and non-tumoral liver tissue) in order to deliver no more than an average of 60 Gy to the whole non-tumoral liver, where the average included the non-injected portion [27], while respecting the manufacturer’s indication of administering less than 150 Gy to the injected portion. The tumor predicted dose was also considered in the choice of the therapeutic activity.

We intentionally considered only patients injected with ^90^Y glass microspheres on Monday of the second week after the calibration date (7.75 days decay interval) in this study. Therapy was administered 3.9 ± 1.7 weeks after the simulation.

### 2.2. Simulation Phase

A total of 150 MBq of ^99m^Tc-MAA was injected for the simulation session (MAASOL by General Electric up to February 2016, then MACROTEC® by Bracco Diagnostics, Milan, Italy). Planar whole-body scintigraphy and SPECT/CT were performed with the two-head Symbia Intevo™ T2 SPECT/CT with a 5/8” NaI crystal thickness by Siemens Medical Solutions, Hoffmann Estates, USA. Hybrid scans were acquired with raised arms for compliant patients (110 kV CT voltage), while they were acquired with the arms along the trunk for non-compliant patients (130 kV CT voltage). No iodinated contrast medium was used. A CT dose reduction was obtained with the CareDose™ system by Siemens Helthcare GmbH, Erlangen, Germany. The CT quality was fixed at a reference of 120 mAs. SPECT images were acquired with an emission energy window centered at 140 keV (20% wide) and with an adjacent lower scatter window with the same width, as well as a 256 × 256 matrix, 3° angular sampling, 60 projections per head, and 20 s per projection in step and shoot mode. The SPECT images for dosimetry were reconstructed with the quantitative Ordered Subsets Conjugated Gradient (OS-CG) algorithm for soft tissues (Siemens xSPECT™ software, by Siemens Medical Solutions, Hoffmann Estates, USA) with 72 iterations, a single subset, and no additional filter. Automated attenuation and scatter corrections inside the iterative loop were applied. Resolution recovery was included in the reconstruction algorithm.

### 2.3. Volume of Interest Definition

Dosimetry was performed on the ^99m^Tc-MAA and ^90^Y PET images. The segmentation was performed with the module of an IMALYTICS™ workstationby Philips Medical Systems, B.V. Best, Nederland. VOIs were drawn on the CT volumes of lesions and on the whole non-tumoral liver tissue on coregistered SPECT/CT images. These were drawn by young physicists (MS, RC, DNMC) with final approval from a nuclear medicine specialist (MM). In case of doubt, interventional radiologists were consulted (SC, MC, MG, GA, CT). In large lesions with a visible necrotic core in CT, the core was excluded from the lesion volume. VOI statistics were exported to a spreadsheet to perform mean absorbed-dose calculations. The spreadsheet is available in the supplementary materials of [13].

### 2.4. Post-Therapy Imaging

For the ^90^Y-PET acquisition, two scanners were used: GEMINI™ 64 TOF by Philips Medical Systems Nederland B.V. Best and DISCOVERY™ 710 TOF by General Electric, Chicago, USA. The acquisition time per bed position was 15 min, with two bed positions in the absence of lung shunt, or 10 min/bed position, with three bed positions covering the lung in the presence of lung shunt at MAA. Patients with lung shunt were preferably scanned on the DISCOVERY 710 to avoid the underestimation by GEMINI reported in the QUEST study [28]. The reconstruction protocols were the Blob-OS-TF algorithm (3 iterations, 33 subsets, smooth and sharp) for Philips and the QClear penalized likelihood algorithm (26 iterations, 48 subsets, noise regularization parameter β = 1500) in the GE case.

The ^90^Y-PET images were coregistered to ^99m^Tc-MAA SPECT images on a e.soft workstation (Siemens Medical Solutions, Hoffmann Estates, USA) with automated rigid coregistration (mutual information algorithm). This allowed us to copy previously defined VOIs onto PET images, thus avoiding inaccuracies derived from having a second VOI definition. Coregistration was always visually inspected.

### 2.5. Voxel Dosimetry

Voxel dosimetry was based on the following assumptions: permanent trapping of microspheres, identical biodistribution for ^99m^Tc-MAA and ^90^Y, and local energy deposition [13,17,29]. Activity quantification was performed through a patient-specific conversion factor given by the ratio of the intended or injected ^90^Y activity and the total counts in SPECT or PET images.

Several dosimetric variables (*Ψ*) were evaluated on pre- and post-therapy images [17]. Aside from the mean absorbed dose (D) for each VOI, radiobiological dosimetric variables such as the equivalent uniform dose (EUD), the average of the biologically effective dose (BEDave) over voxels, and equivalent uniform biologically effective dose (EUBED) were calculated [30]. The radiobiological parameters adopted were the following [17].

For non-tumoral liver: apparent radiosensitivity α’ = 0.002/Gy; α’/β = 10 Gy, T_1/2_^REP^ = 2.5 h.

For lesions: apparent radiosensitivity α’ = 0.003/Gy; α’/β = 10 Gy, T_1/2_^REP^ = 1.5 h.

Moreover, from the cumulative DVH, the non-radiobiological variables D_98_, D_70_, D_50_ (median absorbed dose), and D_2_ were calculated, which were the minimal absorbed doses of 98%, 70%, 50%, and 2% of the VOI, respectively. The homogeneity index (HI) was evaluated as follows [31]:

(1) $$\mathrm{HI}=\frac{D_{2}-D_{98}}{D_{50}}$$

The dosimetric parameters’ estimation and cDVH were computed by a homemade program in MATLAB™ version 7.5, by MathWorks, Natick, USA.

### 2.6. Liver Decompensation Definition

Our toxicity endpoint was the occurrence of treatment-related liver decompensation type C that required medical action (LDC) within six months after TARE, as defined in [17]. The occurrence of LDC, imputability, and irreversibility [32] were assessed by an expert hepatologist (SB).

### 2.7. Response Assessment Methods

The treatment efficacy was investigated by evaluating only measurable lesions that presented a nodular pattern. The mRECIST response assessment method with consensus for HCC was used [23]. In parallel, an additional method was tested, which was an experimental variation of the method of Choi et al. [20]. While in the original paper [20], the cutoff between responding (CR + PR) and non-responding (SD + PD) lesions was at a 15% HU reduction in a circular lesion region of interest (ROI) on the arterial phase (for another kind of tumor and systemic treatment), we adopted here a response threshold at 20% HU variation, as determined in the SPETc-DOSE study. Additional details about the adopted densitometric method are reported in the Supplementary Materials.

Contrast-enhanced multiphasic CT scans were acquired the day before angiography (basal scan). The lesion response was then assessed at every third month post-TARE on the arterial phase of CT follow-up scans by two young radiologists (AG and GM). With both radiological methods, their independent best-response times were considered.

Only lesions with both ^99m^Tc-MAA SPECT and ^90^Y PET image data available were considered. Patients who underwent other post-TARE treatments (loco-regional or systemic treatments, e.g., TACE or sorafenib), as well as non-measurable lesions, were excluded from the dose–response analysis. Only the lesion-by-lesion response (“local response”) was monitored, not the patient’s oncological response.

On these bases, the local objective response (LOR) was defined as the ratio between the sum of the complete response (CR) and partial response (PR) over the total number of lesions analyzed, while the local disease control rate (LDCR) was defined as the sum of the CR, PR, and SD over the total number of lesions. Other oncological efficacy indicators (OS and PFS) were beyond the aims of this study and are not reported here.

### 2.8. Tumor Control Probability (TCP) Curve

To describe the increase in efficacy with lesion dose, i.e., the tumor control probability (TCP) as a function of the lesion’s absorbed dose, two models were adopted. The Poisson model was used when it was applicable according to its definition, i.e., when only complete responses were considered. In this model, the TCP is calculated from the cellular surviving fraction (SF):

(2) $$TCP=e^{-N_{c}\cdot SF}$$

where $N_{c}$ is the total number of clonogenic cells, which depends on the tumor volume ($V_{tumour}$) according to

(3) $$N_{c}=\rho \cdot V_{tumour}\;$$

where ρ is the density of clonogenic cells [33].

The *SF* can be described as a function of the dosimetric parameter *Ψ* from the following equation:

(4) $$SF=e^{-\alpha \cdot \Psi }$$

where *α*′ is the apparent radiosensitivity [17] of the cellular population (1/Gy). The following TCP equation is then returned:

(5) $$TCP=e^{-N_{c}\cdot e^{-\alpha \prime \cdot \Psi }}$$

In the more general case where both CR and PR lesions are considered as responding lesions, the Poisson model cannot be applied. The following empirical log-logistic function was adopted [34]:

(6) $$TCP=\frac{1}{1+{\left(\Psi 50/\Psi \right)}^{k}}$$

where *Ψ50* is the *Ψ*-value at which 50% of tumors respond. k is related to the normalized dose–response gradient (γ) according to *k*= *Ψ*50/(4γ) and controls the curve’s slope.

To obtain the TCP curve, the dosimetric range was divided into three bins. In each bin, the experimental TCP value was the observed response ratio.

### 2.9. Data Analysis and Statistics

The differences between the pre- and post-therapy mean absorbed dose were analyzed with the Bland–Altman method [35].

The correlation between *Ψ* and the response was assessed by modeling and fitting the dose–response curve with the following equation [17]:

(7) $$response=intercept+slope\cdot Log_{10}\left(\Psi \right)$$

The goodness of fit was assessed through the determination of R^2^. The R^2^ was not adapted for the number of parameters. Furthermore, a Spearman correlation test was performed.

To perform a toxicity and efficacy analysis as a function of *Ψ*, dichotomic outcome values were necessary. Patients were divided into two groups according to the presence/absence of LDC. Similarly, lesions were grouped into responding (CR + PR) and non-responding (SD + PD) lesions according to both of the radiological methods adopted.

The agreement between each dosimetric variable *Ψ* and the observed outcome was assessed with four methods: Spearman non-parametric correlation analysis, a median Ψ comparison with a non-parametric Mann–Whitney test, the area under the curve (AUC) of ROC curves, and a tumor control probability curve (TCP).

The ROC analysis was exploited to evaluate the separation in terms of *Ψ* between the true-positive (LDC, responding lesions) and true-negative (no LDC, non-responding lesions) cases. The AUC under the ROC curve measured this separation. The dosimetric variable with the significantly highest value of the AUC should have been considered the best dosimetric descriptor [17].

Since 9 covariates of *Ψ* were used, the problem of Bonferroni’s correction for the repeated significance test was discussed. The strong correlation among the covariates—probably except for the extreme D_98_, D_2_, and HI—would not require Bonferroni’s correction. However, to interpret the results from two opposite points of view (where correction is necessary or not), it was considered that, to keep the usual risk of type I error < 0.05, the *p*-value should be < 0.05 if Bonferroni’s correction is not required, while it should be <0.05/9 = 0.0056 if Bonferroni’s correction is necessary. In our tables, *p*-values < 0.05 are in bold character (significant without Bonferroni’s correction), with an additional “*” if *p* < 0.0056 (significant with Bonferroni’s correction). The correlations among variables were tested (Spearman’s test).

All of these analyses and all fittings were accomplished with the Prism™ software, version 5.03, from GraphPad Software Inc. (San Diego, CA, USA).

## 3. Results

### 3.1. Analyzed Cohort

We initially considered 175 subjects. The median follow-up time according to the reverse Kaplan–Meier estimator was 27.7 months. The mean injected activity in the liver was 2.1 ± 1.1 GBq. In 147 cases, ^90^Y-PET data were available. For the liver decompensation analysis, only Child A patients with at least 6 months of follow-up (FU) data were considered, as in our previous papers (Table 1, second column). The treatment efficacy was investigated by evaluating 106 lesions in 69 patients (Table 1, third column).

For the LDC analysis, imputability and follow-up data were available for 101 patients out of 175, 86 of which presented post-therapy ^90^Y-PET data. Only 4/101 (4%) patients presented LDC (only for three of these patients were ^90^Y-PET data available). Their basal bilirubin was 1.33, 1.96, 1.70, and 1.02 mg/dL, i.e., in 3/4 cases, we had a risk factor (published afterwards) of basal bilirubin of >1.1 mg/dL [27] and one value close to this cut-off value. We consider this toxicity dataset unreliable due to the scarcity of observations and because the bias of bilirubin was greater than 1.1 mg/dL. Though they were performed, the ROC and NTCP analyses are not reported.

### 3.2. Bland–Altman Analysis

For the lesions, we found a bias of −63 Gy and a 95% confidence interval (CI) between −493 and 366 Gy; for the parenchyma, we found a bias of 3 Gy, and 95% CI = [−15 Gy; 22 Gy].

### 3.3. Response Rates

Table 2 presents the local response rate, the local objective response (LOR), and local disease control rate (LDCR); the densitometric method was defined as with mRECIST. Note that the local disease control rate was 97%. Fisher’s exact test did not show any statistically significant difference between the numbers of responding (CR + PR) and non-responding (SD + PD) lesions according to the two radiological methods.

### 3.4. Correlation between Ψ and Response

**Figure 1:**
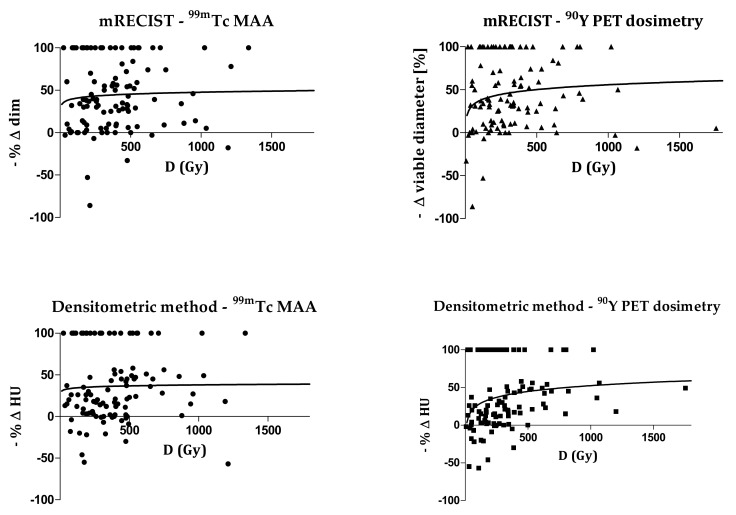
Correlation between mean absorbed dose D and response. The poor correlation can be visually noted. Given a dose value, largely different kind of response were observed, with both methods.

As an example, Figure 1 shows the correlations between the mean dose D and the response. Spearman’s r values for the densitometric method (^99m^Tc-MAA: r = 0.23, *p* = 0.02; ^90^Y-PET: r = 0.43, *p* < 0.0001) are higher with respect to mRECIST (^99m^Tc-MAA: r = 0.09, *p* = 0.37; ^90^Y-PET: r = 0.16, *p* = 0.11). The correlation fit results for all of the studied dosimetric variables are presented in Table 3 and Table 4 with the R^2^ values, Spearman’s r, and *p*-values.

With the mRECIST method, no *p*-value was statistically significant. With the densitometric method, all of the ^90^Y-PET data showed a statistically significant correlation, even with Bonferroni’s correction, except for D_70_. The ^99m^Tc-MAA SPECT data returned statistically significant correlations for D, BED_ave_, D_50_, and D_2_ if Bonferroni’s correction was neglected. No fit convergence was returned for the D_98_ and HI data.

In summary, a better correlation was generally obtained with the densitometric method in comparison with the mRECIST method and for the ^90^Y-PET data in comparison with the ^99m^Tc MAA-SPECT data.

### 3.5. Classes of Responding vs. Non-Responding Lesions

**Figure 2:**
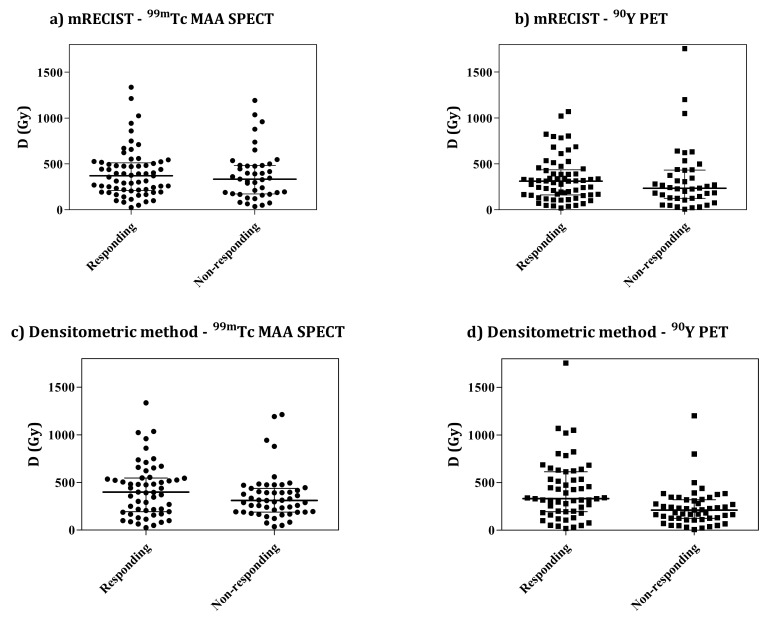
The ^99m^Tc-MAA SPECT/CT (**a**,**c**) and ^90^Y-PET/CT (**b**,**d**) mean dose D distribution for responding and non-responding lesions. Response was evaluated with mRECIST (**a**,**b**) and the densitometric method (**c**,**d**).

Figure 2 presents an example of the distribution of mean dose values of the two classes of responding and non-responding lesions. No evident separation between responding and non-responding classes can be visually highlighted, especially at low absorbed doses, while a weak trend toward a higher absorbed-dose tail for responding lesions is visible. Considering the similar plots shown in Figures S3 and S4 in the Supplementary Materials, the situation is visually worse here—where purely nodular lesions were injected 7.75 days from the calibration date—than there—where mixed lesions were injected at 3.75 days. Here, one can see a number of non-responding lesions at absorbed doses > 500 Gy.

Table 5 (mRECIST) and Table 6 (densitometric method) report the median *Ψ* and *p*-values obtained with the Mann–Whitney test. If we neglect Bonferroni’s correction, a statistically significant difference is obtained only for the ^90^Y-PET data with the densitometric method for all variables of *Ψ*, except for HI. If Bonferroni’s correction is applied, significance is lost for D_98_. In all of the other cases, no significant difference was found. The situation was similar to that of the correlation analysis: Only the densitometric method applied to ^90^Y PET post-therapy dosimetric data was able to provide a significant link between *Ψ* and response.

### 3.6. ROC Analysis

**Figure 3:**
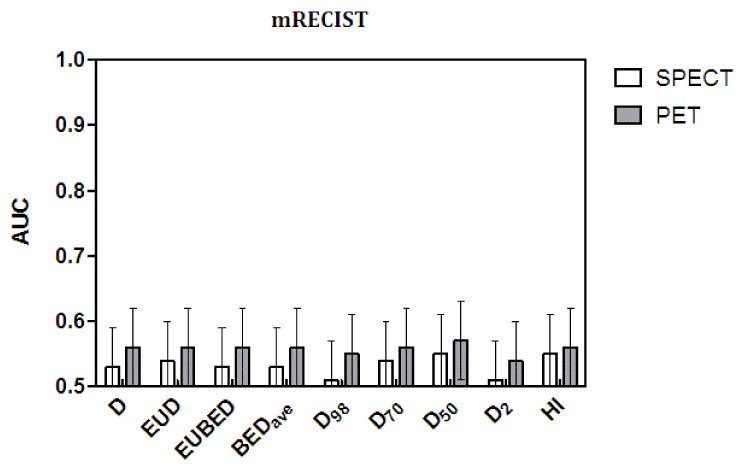
The AUC values for the ROC of ^99m^Tc-SPECT and ^90^Y-PET returned with the mRECIST method.

**Figure 4:**
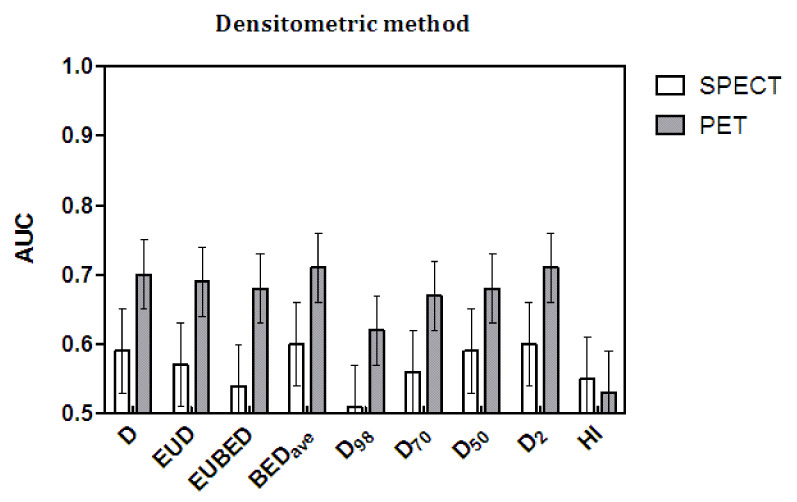
The AUC values of the ROC for ^99m^Tc-SPECT and ^90^Y-PET returned with the densitometric method.

The AUC values and their standard error (SE) values are shown in Figure 3 and Figure 4 as histograms. The numerical values are reported in Table 7 (mRECIST) and Table 8 (densitometric). All of the AUC values were suboptimal by far, as they were remarkably lower than the ideal value of one, with a maximum value of 0.71 obtained with BED_ave_ and with D_2_ (the maximal lesion dose). As for the correlation and Mann–Whitney tests, the statistical significance in the AUC was reached only with the densitometric method applied to post-therapy ^90^Y PET dosimetry.

Regarding the comparison among dosimetric variables, the significance test for differences in AUC by Hanley and McNeil [36] is not necessary for a conclusive interpretation of Figure 3 and Figure 4. A visual check of the AUC values and, above all, of their error bars is enough to conclude that no variable produced a significant improvement in the AUC values with respect to any other or, in particular, with respect to the mean absorbed dose D. On the contrary, HI and, to some degree, D_98_ gave the worst performance with the application of the densitometric method and post-therapy evaluations.

The comparison between response assessment methods is far more interesting. As for the correlations and Mann–Whitney test, the densitometric method also performed significantly better with the AUC and with both pre- and post-therapy data. Comparing as paired data the AUC values obtained with the densitometric method and the mRECIST method, the *t*-test gave *p* = 0.01 for the ^99m^Tc-MAA SPECT data and *p* = 0.0006 for ^90^Y-PET data, with significantly higher AUC values given by the densitometric method.

Finally, once again, the predictions of post-therapy ^90^Y PET were superior to those of MAA. For each radiological response method, the gray bars (^90^Y-PET data) are always higher than the white bars (^99m^Tc-MAA SPECT data)—except for the failure of HI with the densitometric method—with significant *t*-test results for the paired data (*p* < 0.0001 for mRECIST and *p* = 0.0002 for the densitometric method).

### 3.7. Correlations among Variables

**Figure 5:**
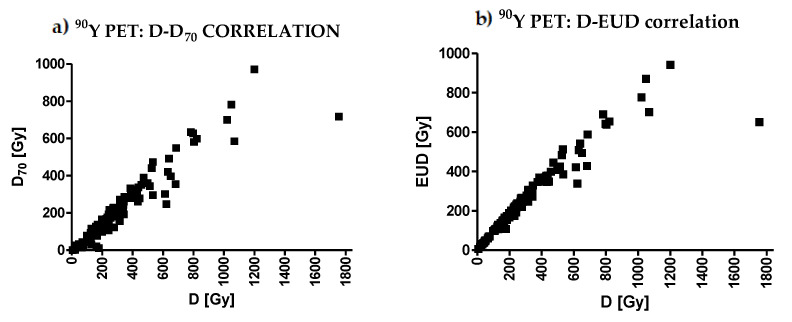
Two visual examples of correlations between the mean lesion absorbed dose D (*X* axis) and (**a**) D_70_ and (**b**) EUD.

Table 9 shows the Spearman correlation coefficients among pairs of dosimetric variables. All *p*-values were <0.0001, except those shown in the last column for HI. With ^99m^Tc-MAA dosimetry, all pairs of variables were correlated with r ≥ 0.67, except for the pair D_98_–D_2_ (minimum dose–maximum dose, r = 0.47) and HI, which is not a dosimetric variable but an estimator of relative non-uniformity. In particular, looking at the first row of values, all variables (except HI) were correlated with the mean dose D, with a minimal but still strong correlation for D_98_ (r = 0.72). With ^90^Y PET dosimetry, the correlations were even stronger. The minimal correlation coefficient was again for the pair D_98_–D_2_ (minimum dose–maximum dose, r = 0.61). Figure 5 shows two examples of correlations: D–D_70_ and D–EUD.

### 3.8. TCP Curve

**Figure 6:**
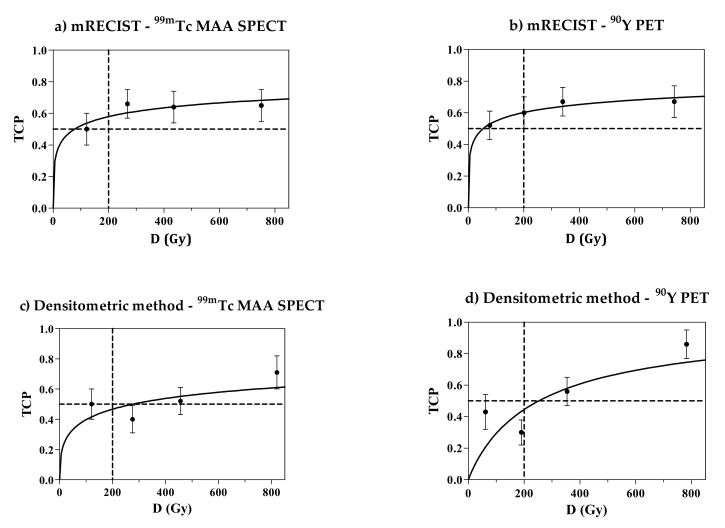
The ^99m^Tc-MAA SPECT (**a**,**c**) and ^90^Y-PET (**b**,**d**) Tumour Control Probability (TCP) curves for the empirical log-logistic function fitting curves as function of the mean dose D (equation 6). The results of the mRECIST (**a**,**b**) and of the densitometric method (**c**,**d**) are presented. The dashed lines are a grid at 200 Gy and TCP = 50% to improve visual comparisons between different plots.

In Figure 6, the TCP curves obtained for the mean dose D with the empirical log-logistic function (Equation (6)) are shown (106 lesions, CR and PR lesions considered as responding lesions).

Note that according to mRECIST, the response probability seems to have a plateau that is far lower than 1. In other words, there is no gain in response probability above 300 Gy and, presenting a serious problem, the certainty of response is not reached up to 800 Gy. For the densitometric method, the trend of TCP at high doses is slightly steeper and closer to the expected value. However, the chance of response is still less than 1 at 800 Gy when evaluated with the more reliable ^90^Y-PET images (lower-right panel in Figure 6).

**Figure 7:**
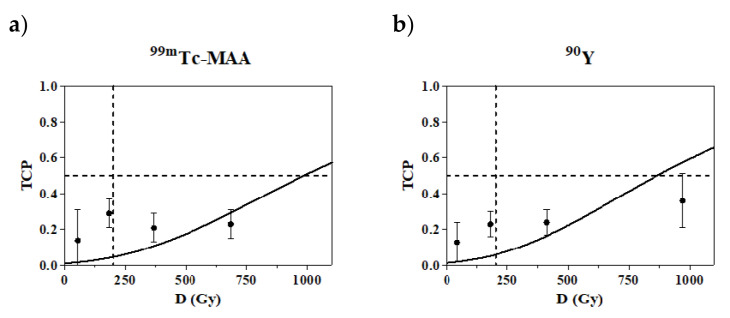
The Poisson model-fitting curves for the ^99m^Tc-MAA SPECT (**a**) and ^90^Y-PET (**b**) Tumour Control Pobability (TCP) curves (Equation (5)) with Complete Response (CR) as endpoint. Lesions with CR were the same with the two methods. The dashed lines are only a grid to improve visual comparison between different plots.

In Figure 7, only the CR is considered (mRECIST only). The TCP curves obtained with the Poisson model are presented as a function of the mean absorbed dose D. The passage at the axis’s origin (0, 0) was imposed. Despite that, the experimental data are badly fitted by the Poisson model. Apart from the model, we observe again that the experimental TCP values do not increase with the absorbed dose. The α’ values obtained from the fit are 0.002 ± 0.001/Gy for both pre- and post-therapy dosimetry.

### 3.9. Disease Control Rate

The local disease control rate (LDCR) obtained with the mRECIST criterion (97%) is very satisfactory. This means that only 3% of the treated lesions underwent a progression. However, these data do not cover non-measurable lesions, which mainly account for infiltrative tumors. Moreover, possible progression of lesions in the non-injected liver lobe was not considered. Therefore, the reported local response evaluation on only measurable target lesions might be excessively optimistic with respect to the usual patient-based oncological evaluations.

### 3.10. Pre- Versus Post-Therapy Dosimetry

The worse results obtained in general with ^99m^Tc-MAA SPECT/CT compared to ^90^Y PET dosimetry are explained by the Bland–Altman analysis of the differences between post- and pre-therapy dosimetry. It is known that the predictive dosimetric accuracy of ^99m^Tc-MAA is sometimes suboptimal for lesions [37].

### 3.11. No Improvement Using Voxel Dosimetry in TARE

Unexpectedly, in this study, none of the dosimetric variables *Ψ* offered a significant improvement in the agreement with the response data with respect to the mean absorbed dose. The results obtained in our previous paper are confirmed [17], although we solved all previous flaws and we considered additional variables that were independent of the radiobiological parameters (D_98_, D_70_, D_50_, D_2_). A similar result was reported by Dewaraja et al. [38] and by Kappadath et al. [39]. A different conclusion was found for complete response with resin microspheres by Kao et al. (D_70_ > 100 Gy) [40]

The strong correlations between all dosimetric variables (except HI) and the mean dose D explains this fact. The reason for these correlations could be the limited spatial resolution of nuclear medicine images, which blurs non-uniformity at the voxel level, thus acting as a smoothing filter. The beta energy transport among voxels and the breathing motion contribute to this degradation of image resolution. This hypothesis, which has serious consequences for voxel analysis in nuclear medicine, could and should be verified using virtual images with ideal resolution.

### 3.12. Dose–Response Relationship

#### 3.12.1. The Importance of the Radiological Response Assessment Method

Evaluation of the radiological response of HCC to TARE is a tough task. According to our preliminary study (Supplementary Materials), the densitometric method was applicable in 97% of lesions, while mRECIST was applicable in a reduced percentage (89%). In the present study, the agreement obtained with the densitometric method with dosimetry overcame mRECIST in terms of the dose–response correlation, Mann–Whitney tests, and AUC comparison. We demonstrated how the quality of the dose–response relationship depends on the radiological method adopted, not only on the accuracy of the dosimetric variable. Our results bring into the discussion the appropriateness of the mRECIST method in TARE of HCC and support the promising densitometric method, as already proposed by Gavanier et al. [22].

The Liver Cancer Study Group of Japan published another method for assessing the direct effects of treatment on hepatocellular carcinoma (HCC) with locoregional therapies, which they called the Response Evaluation Criteria in Cancer of the Liver (RECICL) [41]. Their basic idea was the same as that of the present work: Necrotization of treated lesions should be accounted for by a response assessment method. The RECICL is, however, still based on a bi-dimensional orthogonal diameter evaluation that is taken in the arterial phase in order to derive the extension of the necrotized area after therapy. However, the degree of necrotization is not measured in terms of the difference in Hounsfield units (HUs). Tissue is considered as viable or necrotic with a sort of digital “yes/no” assessment of necrotization, as mRECIST and EASL consider tissue as viable or not and enhanced or non-enhanced. On the contrary, the densitometric method focuses on a quantitative measurement of the degree of necrotization with HUs considered as a continuous variable. We believe that this is a key point in the evaluation of the response of HCC to TARE. This seems confirmed by the much better agreement of the HU difference with the dosimetric data in comparison to the dimensional criterion of mRECIST.

We remark that the present work is not a clinical validation of the densitometric method with the threshold shifted to 20%. The unexpected significantly improved agreement with the dosimetric data is probably supported by basic reasons. Differently from other tumors and treatments, HCC treated with TARE seldom shrinks, but more often reduces its density, at least initially. The densitometric criterion may therefore be more suitable. We believe that it deserves further investigation and clinical validation.

As a summary of the three major aspects discussed above, we state the major conclusion of our work: The best dose–response relationship did not require a variable other than the mean lesion absorbed dose, which was evaluated on post-therapy ^90^Y PET images by assessing the response with the densitometric method with the threshold at 20%. However, other collateral observations deserve discussion.

#### 3.12.2. Poor Dose–Response Correlation and Poor Separation between Responding and Non-Responding Lesions

The dose–response correlation obtained was poor for every variable *Ψ* considered. Even in the best case, the densitometric method with post-therapy values and D_50_ gave a low Spearman’s r of 0.46 and R^2^ = 0.17. This makes it difficult to predict response in individual cases. In our previous work [17], we obtained better R^2^ values—between 0.34 and 0.4—but by using an excessive 50% threshold for the densitometric response assessment method and about one-third of the number of microspheres per GBq.

Similarly, for all of the variables *Ψ* considered, the AUC values for lesions were low. The maximum AUC value obtained was 0.71. This was reported also by Kappadath et al. (AUC = 0.72 for both mean absorbed dose and mean BED), who studied HCC with post-therapy ^90^Y glass microsphere bremsstrahlung with SPECT/CT [39]. The values obtained by Dewaraja et al. were higher: AUC between 0.88 and 0.90 [38]. However, they studied smaller lesions. Their median tumor mass was about 10 g, while we had a median of 56 g. Their patients showed heterogeneous tumor types and were injected at variable decay intervals from the calibration date. In our present study, this was fixed at 7.75 days. They evaluated response only in the first follow-up CT scans, while we chose the best response time. All of these differences make the comparison difficult.

The crucial problem regarding the dose–response relationship is visually evident in the dose distributions in Figure 2 of this main text and in Figures S3 and S4 of the Supplementary Materials. The dose distributions of responding and non-responding lesions overlap. A trend toward separation is found only in the high dose tails, and not at low doses. Moreover, responses at low absorbed doses were common with both radiological methods. Similar plots were reported by Kappadath et al. and by Dewaraja et al. [38,39]. Responses at relatively low absorbed doses could have an important meaning: A low absorbed dose could be effective if optimal conditions met (good microsphere distribution at the microscopic scale). On the contrary, a high absorbed dose could not be effective if such conditions are not met (bad distribution at the microscopic scale). Unfortunately, the absorbed-dose distribution pattern at the microscopic scale is inaccessible with the present spatial resolution of the images. From the above-mentioned figures, we therefore have the immediate practical drawback that overlapping dose intervals make it difficult to fix a reliable and reasonable efficacy threshold. At a deeper level, it seems that response depends on an additional inaccessible variable (dose distribution at the microscopic scale), which is in agreement with Dewaraja et al. [38], and is linked to the number of microspheres/GBq.

In both of our works and in Dewaraja et al. [38], the correlations between the dose and response with microspheres are markedly worse (R^2^ = 0.14 for mRECIST, R^2^ =0.14 for the densitometric method) than those obtained with the radiopharmaceutical ^177^Lu-DOTATATE (R^2^ = 0.64 if diameter > 2.2 cm, R^2^ = 0.91 if diameter > 4 cm) [42]. The only hypothesis to explain this refers to the different dose deposition patterns at the microscopic scale. ^177^Lu-DOTATATE is actively taken up by all cells with somatostatin receptors on their membranes. Microspheres are non-uniformly deposited inside micro-capillaries and do not uniformly reach all tumor cells. Moreover, microspheres are known to cluster [43]. This further deteriorates the uniformity of the dose deposition at the microscopic scale.

Another possible reason for the markedly different degrees of response with the same absorbed dose might pertain to biology. Variable radiosensitivities of individual tumors are certainly present, as confirmed by the dispersion of the radiosensitivity values obtained (α’ = 0.002 ± 0.001/Gy). It is, however, strange that variable tumor radiosensitivity could worsen the correlations in HCC treated with microspheres so heavily despite the high absorbed doses delivered, but not in neuro-endocrine tumors treated with ^177^Lu DOTATATE. Therefore, we think that the main problem causing the poor dose–response correlations with microspheres is the inaccessibility to the absorbed-dose distribution at the microscopic scale, rather than the variability in radiosensitivity.

Whichever is the reason for the poor dose–response correlation shown in Figure 1, it seems that an additional factor decreases the reliability of the dose-based tumor response predictions with microspheres with respect to radiopharmaceuticals, together with the limited accuracy of the MAA absorbed-dose prediction for lesions. This pushes us toward the maximum tolerable dose approach, which is based on the non-tumoral whole-liver dose [27].

#### 3.12.3. Confirmation of the Much Lower Apparent Radiosensitivity than in External Beam Radiotherapy

The lesion radiosensitivity α’ = 0.002 ± 0.001/Gy obtained with the TCP curve fitting considering only CR confirmed our previous value of 0.003/Gy [17] and those from Strigari et al. for resin spheres (α’ = 0.001/Gy and α’ = 0.005/Gy) [44]. Dewaraja et al. obtained even lower values in the range 0.0001–0.002/Gy [38]. This “apparent” radiosensitivity value, which is five times lower than that obtained in EBRT (α = 0.01 ± 0.001/Gy) [45], is explained by the lower uniformity of irradiation at the microscopic scale with respect to external beams [17].

#### 3.12.4. Bad TCP Curve Behavior

For almost all of the TCP curves (Figure 5), it is evident that a probability plateau is reached well below 1.00, around 0.75. This means that, even for highest absorbed doses delivered, the tumor response is not reached for all lesions. The CR rate reported from the Poisson model-fitting curves (Figure 6) reached a value of only 20%, and did not increase despite the increase in absorbed dose. In our previous work [17], the TCP curve for CR + PR reached the value of 100%, but when using the 50% HU threshold for response. This more demanding threshold should have produced a lower TCP curve, but here, we observed the opposite phenomenon. Another factor should be invoked to explain this reduced efficacy at high doses. This can only be linked to the higher number of microspheres/GBq used in the present study. However, the comparison between the dose–response relationships obtained here and in [17] is biased by the methodological differences between the two works, as described in the introduction. The study of the influence of the number of glass microspheres/GBq requires a cohort comparison where the same imaging and dosimetric methods will be applied to both sides (few and many spheres/GBq), as well the same response assessment method.

#### 3.12.5. The Hypothesis of Mega-Cluster Formation

We reported a worse dose–response correlation, a worse separation between responding and non-responding lesions, and, above all, a worse TCP trend than in [17] as collateral observations. Two major methodological differences might explain these facts. The first is the lower threshold for HU response with the densitometric method (20% versus 50% [17]). This is excluded, since all of these observations were also obtained with mRECIST, though this method was not used in [17]. The other difference was the injection day and the consequent number of microspheres/GBq used in the present work that was 2.8 times higher. However, according to our previous considerations [46], an increased number of microspheres should have improved efficacy, though we observed a worsening.

The only possible hypothesis to explain this dilemma is derived from the study by Högberg et al. [47], which was performed on an explanted human liver portion treated with resin spheres. They assessed the real particle spatial distribution on microscopic level ex vivo and evaluated the resulting absorbed dose distribution. The main result was that the frequency of formation of mega-clusters increased exponentially with the increase in the mean local absorbed dose. This produced a worsening of the uniformity of the absorbed-dose deposition at the microscopic scale in regions with a higher mean local absorbed dose. This implies a reduction of the biological effects (toxicity and efficacy).

However, the work by Högberg et al. was performed with resin microspheres and might not be applicable to the present study, which was performed with glass microspheres. Indeed, in a similar study of four explanted livers, Kennedy et al. [43] reported the clustering phenomenon and that “resin and glass microspheres dispersed similarly in the liver”.

The more recent paper by Pasciak et al. [48] is closer to our situation, since they administered glass microspheres in livers of pigs, which are the most similar to human livers. A 50 Gy mean absorbed dose was delivered to the non-tumoral liver lobe with injections 4, 8, 12, and 16 days after the calibration date. They observed a progressive reduction of the coefficient of variation (CV) of the absorbed dose among voxels with the progressive increase in the number of microspheres per GBq: CV = 4.77, 2.32, 1.25, and 1.28, respectively. Therefore, the decrease in the CV apparently produced an increase in the uniformity in the absorbed-dose deposition at the microscopic scale and an augmented biological effect (toxicity, according to the authors). Their conclusions seem opposite to our data. Note, however, the reverse trend in the last figure, an increase from 1.25 to 1.28, passing from 12 to 16 days. A finer argument is necessary.

For the first time, Pasciak et al. [48] provided a valuable direct measurement of the true mean specific activity per glass microsphere: 4353.8 Bq at the calibration date (not 2500 Bq, as reported in the manufacturer’s manual). Let us assume the tissue density = 1 g/cm^3^. Let us express the injected activity as A = N × V × a, with “a” (activity per microsphere) times N, the number of microspheres per cm^3^, times V, the injected volume. Using the known formula D = 50 A/M, 50 Gy are delivered by injecting 1 GBq into 1 kg. We can easily compute that 50 Gy correspond to a spatial density of N = 1 MBq/4353.8 Bq/1 cm^3^ = 230 microsphere/cm^3^ at the calibration date. Following Pasciak et al., in order to deliver a fixed mean absorbed dose of 50 Gy, if we inject with 4-day decay intervals, we need N = 651, 1842, 5213, and 14753 microspheres/cm^3^ on days 4, 8, 12, and 16, respectively, with a = 1541, 546, 193, and 68 Bq/microsphere, respectively.

We point out the most important phenomenon reported by Pasciak et al.: a sudden jump in cluster size from five particles/cluster on days 4, 8, and 12 to 13 particles/cluster (mega-cluster) only on day 16. This is a non-linear phenomenon that is a function of the particle spatial density, which is somehow compatible with the exponential increase in mega-cluster size noted by Hogberg et al. We could say that with more than N_critical_ of about 15,000 glass microspheres/cm^3^, the mega-cluster phenomenon is triggered. By injecting glass microspheres 7.75 days after the calibration time, a = 582 Bq/microsphere, and N_critical_ corresponds to a critical absorbed dose of about D = 50 A/M = 50 N_critical_ × V × a/M = 437 Gy.

Therefore, in tumors with doses higher than about 440 Gy injected on day 7.75, the mega-cluster regimen might be triggered. This would reduce the uniformity of the dose deposition pattern at the microscopic scale. Consequently, the efficacy might not increase while increasing the mean absorbed dose, as reported in our TCP plots (Figure 6).

The above argument should not be applied to resin spheres given the rheological differences. The clinical confirmation of the mega-cluster hypothesis for glass microspheres (reduced efficacy for absorbed doses higher than a critical value, depending on the injection day) requires a well-designed study, where the two sides treated on two different days should be studied with the same methodology (dose calculation method and response assessment method).

### 3.13. Limitations of the Present Study

#### Merging Data Obtained with Two Different PET Scanners

The QUEST study reported the underestimation of ^90^Y activity by the Philips GEMINI scanner at concentrations of 3.3 GBq/10 L = 0.33 GBq/L and lower [28]. This concentration corresponds to less than 16.5 Gy. The mean dose to the lobe between 100 and 150 Gy (manufacturer indications) in our patients is one order of magnitude higher. This excludes the underestimation of GEMINI in the liver. In addition, differently from the QUEST study, we adopted the relative patient calibration method, which, by definition, recovers the true total injected activity [13].

The partial volume effect is a general limitation of SPECT/PET quantification. For this specific application, evaluations of spheres with diameter < 2 cm (volume < 4.2 cm^3^) are considered prone to large errors and are discouraged [13,17,28]. We decided not to apply PVE correction based on recovery coefficients. The effect depends on the radial position of each voxel inside the sphere; the correction cannot be applied to voxels.

**Figure 8:**
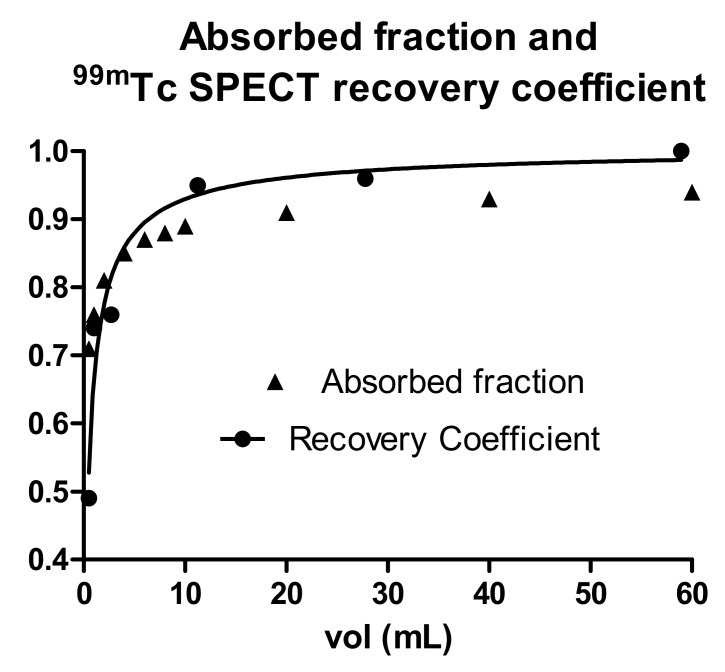
Dependence of the absorbed fraction and recovery coefficients on the sphere volume.

Moreover, the dependences upon sphere size of the absorbed energy fraction and of the recovery coefficients are similar. Figure 8 shows the absorbed energy fraction for water spheres calculated with the IDAC software and the recovery coefficient of ^99m^Tc spheres in a water phantom, obtained with a Siemens Symbia Intevo T6 with a 256 × 256 matrix, OS-CG reconstruction, 72 iterations, one subset, and no additional filters. The two curves approximately overlap. Then, the two effects cancel out each other, with no need for PVE correction for the mean dose evaluation.

This study did not include more refined clinical parameters, such as variable individual radiosensitivity linked to the genetic profile. In addition, multivariate analyses with the absorbed dose and lesion volume as covariates might be useful.

## 4. Conclusions

Despite having removed all of the methodological flaws present in our previous study, for the second time, and in agreement with other authors, voxel dosimetry did not improve the interpretation of responses with respect to the mean absorbed dose. This is due to the strong correlation between each voxel variable and the mean absorbed dose, which is probably caused by the limited spatial resolution of nuclear medicine images.

The dose–response correlations, Mann–Whitney tests, and AUC values were statistically significant only when using the experimental and non-validated densitometric radiological criterion with a response threshold at 20% and only with post-therapy ^90^Y PET dosimetry. This encourages further clinical validation of the densitometric criterion in TARE of HCC.

The dose–response correlations and separation in terms of absorbed dose between responding and non-responding lesions were poor. This is attributed to the impossibility of knowing the absorbed-dose distribution at the microscopic scale. This, together with the limited accuracy of MAA predictions observed for lesions, reduces the reliability of planning TARE based mainly on tumor dosimetry in favor of non-tumoral liver dosimetry.

With respect to our previous findings, we collaterally observed a worse dose–response correlation, a separation in terms of absorbed dose between responding and non-responding lesions, and, above all, a decreased efficacy even at 800 Gy with both response assessment methods. These phenomena might be imputed to the later injection (day 7.75 versus 3.75 from the calibration date) causing a number of microspheres/GBq that was 2.8 times higher, with a consequent reduction of dose distribution uniformity on the microscopic scale caused by the formation of mega-clusters at high doses. A comparative study with the same methodology is necessary to confirm the clinical impact of the mega-cluster hypothesis.

## Figures and Tables

**Table 1:** Characteristics of the studied patients.

	Initial Cohort	Toxicity Analysis	Efficacy Analysis
No. of patients	175 (100 %)	101	69 (100%)
Age, years mean (range)	67 (27–88)	67 (28–88)	66 (27–87)
**Sex**			
Female	27 (15%)	17 (17%)	10 (15%)
Male	148 (85%)	84 (83%)	59 (85%)
**Etiology of Liver Disease**			
HBV	26 (15%)	17 (17%)	10 (15%)
HCV	87 (50%)	47 (47%)	27 (39%)
HBV & HCV	4 (2%)	3 (3%)	1 (1%)
Other	58 (33%)	34 (33%)	31 (45%)
**Total Basal Bilirubin (mg/dL)**			
Minimum	0.23	0.26	0.26
25% Percentile	0.57	0.5	0.54
Median	0.81	0.77	0.76
75% Percentile	1.27	1.16	1.15
Maximum	6.48	2.62	6.48
**Child Pugh Score**			
A5–A6	159 (91%)	101 (100%)	63 (91%)
B7	16 (9%)	0 (0%)	6 (9%)
**ALBI Score**			
G1	87 (50%)	58 (57%)	36 (53%)
G2	85 (49%)	43 (43%)	32 (46%)
G3	1 (1%)	0 (0%)	0 (0%)
N.A.	1 (1%)	0 (0%)	1 (1%)
**BCLC stage**			
A	9 (5%)	7 (7%)	6 (9%)
B	82 (47%)	51 (50%)	41 (59%)
C	81 (46%)	42 (42%)	22 (32%)
N.A.	3 (2%)	1 (1%)	0 (0%)
**Tumor Distribution**			
Unilobar	96 (55%)	62 (62%)	39 (57%)
Bilobar	79 (45%)	39 (38%)	30 (43%)
**Tumor Pattern**			
Nodular (N)	85 (49%)	56 (55%)	46 (67%)
Infiltrative (I)	32 (18%)	14 (14%)	1 (1%)
N/I	58 (33%)	31 (31%)	22 (32%)
**Number of Nodules**			
1	50 (29%)	33 (33%)	19 (28%)
02–mar	33 (19%)	23 (23%)	18 (26%)
> 3	92 (53%)	45 (44%)	32 (46%)
**Tumor Mass (g)**			
Minimum	1	1	1
25% Percentile	9	10	9
Median	60	50	56
75% Percentile	184	167	142
Maximum	4871	1447	1447
**Tumor Burden**			
< 50%	164(4%)	101 (100%)	68 (99%)
> 50%	11 (6%)	0 (0%)	1 (1%)
**AFP (UI/mL)**			
Minimum	1	1	1
25% Percentile	8	7	6
Median	85	55	18
75% Percentile	1496	658	319
Maximum	400,000	117,431	117,431
**Previous Treatments**			
None	76 (43%)	46 (45%)	34 (49%)
Sorafenib	9 (5%)	0 (0%)	3 (4%)
RF ablation	6 (3%)	4 (4%)	2 (3%)
Liver resection	5 (3%)	1 (1%)	1 (1%)
TACE	34 (19%)	25 (25%)	14 (21%)
Multiple treatment	45 (26%)	25 (25%)	15 (22%)
**Concomitant Treatments**			
Sorafenib	9 (5%)	0 (0%)	0 (0%)
PVT			
Yes	84 (48%)	43 (43%)	21 (30%)
No	91 (52%)	58 (57%)	48 (70%)
**PVT Classification**			
I	39 (46%)	26 (26%)	11 (16%)
II	15 (18%)	8 (8%)	5 (7%)
IIIa	13 (15%)	9 (9%)	4 (6%)
IIIb	16 (19%)	0 (0%)	1 (1%)
IV	0 (0%)	0 (0%)	0 (0%)
**Portal Hypertension**			
Yes	114 (65%)	65 (64%)	45 (65%)
No	61 (35%)	36 (36%)	24 (35%)

*Liver decompensation type C (non-spontaneously reversible liver decompensation, LDC).*

**Table 2:** Response class rate, LOR, and LDCR for lesions evaluated with mRECIST and the densitometric method. The local analysis disregarded possible progression in non-target lesions or due to the appearance of new lesions.

	mRECIST (*n* = 106)	Densitometric Method (*n* = 106)
CR	25 (24%)	25 (24%)
PR	40 (40%)	35 (33%)
SD	38 (38%)	45 (41%)
PD	3 (3%)	3 (3%)
CR + PR	65 (61%)	55 (52%)
SD + PD	41 (39%)	51 (48%)
LOR	61%	52%
LDCR	97%	97%

**Table 3:** R^2^, Spearman’s r, and *p*-values obtained with the ^99m^Tc-SPECT and ^90^Y-PET data for the *Ψ*–mRECIST response correlation evaluation (Equation (7)).

mRECIST	^99m^Tc-SPECT			^90^Y-PET		
*Ψ*	R^2^	Spearman’s r	*p*-Value	R^2^	Spearman’s r	*p*-Value
D	0.00	0.09	0.37	0.04	0.16	0.11
EUD	0.01	0.11	0.27	0.04	0.17	0.08
EUBED	0.01	0.09	0.33	0.05	0.17	0.08
BED_ave_	0.00	0.07	0.48	0.03	0.15	0.13
D_98_	NA	0.06	0.58	NA	0.16	0.10
D_70_	0.01	0.12	0.23	0.04	0.18	0.07
D_50_	0.01	0.13	0.19	0.04	0.17	0.08
D_2_	0.00	0.00	0.97	0.02	0.10	0.30
HI	0.00	0.09	0.37	0.04	0.16	0.11

**Table 4:** R^2^, Spearman’s r, and *p*-values obtained with the ^99m^Tc-SPECT and ^90^Y-PET data for the *Ψ*–densitometric method response correlation evaluation (Equation (7)).

DENSITOM.	^99m^Tc-SPECT			^90^Y-PET		
*Ψ*	R^2^	Spearman’s r	*p*-Value	R^2^	Spearman’s r	*p*-Value
D	0.02	0.23	**0.02**	0.14	0.43	<0.0001 *
EUD	0.01	0.19	0.06	0.13	0.41	<0.0001 *
EUBED	0.00	0.14	0.17	0.12	0.39	<0.0001 *
BED_ave_	0.02	0.24	**0.01**	0.16	0.44	<0.0001 *
D_98_	0.00	0.17	0.07	0.09	0.38	<0.0001 *
D_70_	NA	−0.02	0.84	NA	0.26	**0.01**
D_50_	0.03	0.23	0.02	0.17	0.46	<0.0001 *
D_2_	0.01	0.24	**0.01**	0.12	0.40	<0.0001 *
HI	0.02	0.23	**0.02**	0.14	0.43	<0.0001 *

*p*-values < 0.05 are in bold character (significant without Bonferroni’s correction), An additional * means *p* < 0.0056 (significant with Bonferroni’s correction), significant results obtained in bold.

**Table 5:** Median *Ψ* values (in gray, except HI) and Mann–Whitney *p*-values for responding and non-responding lesions classified according to the mRECIST criterion.

	^99m^Tc-SPECT			^90^Y-PET		
*Ψ*	Responding	Non-Responding	*p*-Value	Responding	Non-Responding	*p*-Value
D	369	333	0.56	311	234	0.31
EUD	309	315	0.49	262	216	0.28
EUBED	475	484	0.62	374	318	0.28
BED_ave_	728	646	0.61	574	422	0.32
D_98_	106	113	0.92	57	46	0.35
D_70_	258	251	0.48	192	169	0.27
D_50_	333	321	0.37	268	223	0.25
D_2_	612	641	0.88	618	582	0.49
HI	2	2	0.32	2	2	0.31

**Table 6:** Median *Ψ* values (in gray, except HI) and Mann–Whitney *p*-values for responding and non-responding lesions classified according to the densitometric criterion.

	^99m^Tc-SPECT			^90^Y-PET		
*Ψ*	Responding	Non-Responding	*p*-Value	Responding	Non-Responding	*p*-Value
D	398	309	0.10	331	210	0.0004*
EUD	367	288	0.24	299	174	0.0010*
EUBED	586	426	0.45	456	232	0.0020*
BED_ave_	798	565	0.08	672	337	0.0003*
D_98_	97	122	0.82	63	41	0.0349*
D_70_	314	226	0.28	256	124	0.0027*
D_50_	390	298	0.10	322	175	0.0013*
D_2_	724	582	0.09	816	467	0.0002*
HI	2	2	0.27	2	2	0.57

*p*-values < 0.05 are in bold character (significant without Bonferroni’s correction), An additional * means *p* < 0.0056 (significant with Bonferroni’s correction), significant results obtained in bold.

**Table 7:** AUC values obtained with mRECIST. No *p*-value reached statistical significance.

mRECIST	^99m^Tc-SPECT				^90^Y-PET			
*Ψ*	AUC	SE	95% C.I.	*p*-Value	AUC	SE	95% C.I.	*p*-Value
D	0.53	0.06	[0.42, 0.65]	0.55	0.56	0.06	[0.44, 0.67]	0.31
EUD	0.54	0.06	[0.43, 0.66]	0.48	0.56	0.06	[0.45, 0.68]	0.28
EUBED	0.53	0.06	[0.41, 0.66]	0.61	0.56	0.06	[0.45, 0.68]	0.28
BED_ave_	0.53	0.06	[0.42, 0.64]	0.60	0.56	0.06	[0.44, 0.67]	0.32
D_98_	0.51	0.06	[0.39, 0.63]	0.92	0.55	0.06	[0.44, 0.67]	0.34
D_70_	0.54	0.06	[0.42, 0.66]	0.48	0.56	0.06	[0.45, 0.68]	0.27
D_50_	0.55	0.06	[0.44, 0.67]	0.37	0.57	0.06	[0.45, 0.68]	0.25
D_2_	0.51	0.06	[0.39, 0.62]	0.87	0.54	0.06	[0.42, 0.66]	0.49
HI	0.55	0.06	[0.44, 0.67]	0.35	0.56	0.06	[0.44, 0.67]	0.33

**Table 8:** AUC values obtained with the densitometric method. The *p*-values are statistically significant for post-therapy evaluations for all dosimetric variables, except for HI.

mRECIST	^99m^Tc-SPECT				^90^Y-PET			
*Ψ*	AUC	SE	95% C.I.	*p*-Value	AUC	SE	95% C.I.	*p*-Value
D	0.59	0.06	[0.48, 0.70]	0.10	0.70	0.05	[0.60, 0.80]	0.0004*
EUD	0.57	0.06	[0.46, 0.68]	0.24	0.69	0.05	[0.58, 0.79]	0.0010*
EUBED	0.54	0.06	[0.43, 0.65]	0.45	0.68	0.05	[0.57, 0.78]	0.0010*
BED_ave_	0.60	0.06	[0.49, 0.71]	0.08	0.71	0.05	[0.61, 0.81]	0.0003*
D_98_	0.51	0.06	[0.40, 0.62]	0.82	0.62	0.05	[0.51, 0.73]	0.0349*
D_70_	0.56	0.06	[0.45, 0.67]	0.28	0.67	0.05	[0.57, 0.77]	0.0027*
D_50_	0.59	0.06	[0.48, 0.70]	0.10	0.68	0.05	[0.58, 0.78]	0.0010*
D_2_	0.60	0.06	[0.49, 0.70]	0.09	0.71	0.05	[0.61, 0.81]	0.0002*
HI	0.56	0.06	[0.44, 0.66]	0.34	0.53	0.06	[0.42, 0.64]	0.54

*p*-values < 0.05 are in bold character (significant without Bonferroni’s correction), An additional * means *p* < 0.0056 (significant with Bonferroni’s correction), significant results obtained in bold.

**Table 9:** Spearman correlation coefficients r among pairs of variables. All *p*-values were < 0.0001 except those for HI, which are reported in the last column.

^99m^Tc-SPECT										
	D	EUD	EUBED	BED_ave_	D_98_	D_70_	D_50_	D_2_	HI	HI *p*-value
D		0.97	0.94	0.99	0.72	0.94	0.99	0.90	−0.28	0.004
EUD			0.99	0.95	0.82	0.99	0.98	0.82	−0.42	
EUBED				0.91	0.88	0.99	0.95	0.76	−0.49	
BEDave					0.67	0.91	0.97	0.94	−0.20	0.038
D98						0.84	0.74	0.47	−0.72	
D70							0.96	0.75	−0.49	
D50								0.85	−0.35	
D2									0.06	0.518
HI										
^90^Y-PET										
	D	EUD	EUBED	BED_ave_	D_98_	D_70_	D_50_	D_2_	HI	HI *p*-value
D		0.99	0.98	1.00	0.81	0.97	0.99	0.92	−0.35	0.0003
EUD			1.00	0.98	0.86	0.99	0.99	0.88	−0.43	
EUBED				0.97	0.88	0.99	0.99	0.86	−0.47	
BEDave					0.77	0.94	0.97	0.95	−0.28	0.003
D98						0.89	0.84	0.61	−0.69	
D70							0.99	0.81	−0.53	
D50								0.86	−0.44	
D2									−0.03	0.780
HI										

## Data Availability

Ethical, legal, and privacy issues prevent single-patient data publication. The retrospective nature of the present study prevented the collection of patients’ consent to publish their own individual data. The research nature of our institution and the approval by the Ethics Committee allowed the retrospective collection such data and their publication as collective information.

## Supplementary Materials

The following report is available online at https://www.mdpi.com/article/10.3390/cancers14040959/s1: Tuning the densitometric radiological response assessment method - Results from the previous study SPETc-DOSE-1 (INT 99-17). This was the part of the work funded by Boston Scientific Corporation.
