# Supplementary material for: Radioembolization of Hepatocellular Carcinoma with 90Y Glass Microspheres: No Advantage of Voxel Dosimetry with Respect to Mean Dose in Dose–Response Analysis with Two Radiological Methods
Source: Cancers (Basel). 2022 Feb 15;14(4):959. doi: 10.3390/cancers14040959 (PMC8869948; doi:10.3390/cancers14040959)
Supplement: Supplementary file 1 [file cancers-14-00959-s001.zip › cancers-1523648-supplementary.pdf]

cancers MDPI

Tuning the densitometric radiological response assessment method

Results from the previous study SPETc-DOSE-1 (2017)

TABLE OF CONTENT

1. INTRODUCTION 1
2. METHODS 1
   2.1 Densitometric method: search for the best density variation threshold 1
   2.2 Search for the best lesion absorbed dose threshold 2
3. RESULTS 3
   3.1 The best response criterion and the optimal dose threshold 3
      3.1.1 Lesion volumes and ^{99m}Tc MAA predicted absorbed doses 3
      3.1.2 Best density variation threshold with the densitometric method 3
      3.1.3 Best dose threshold in densitometric criteria and mRECIST 4
      3.1.4 Analysis of both cohorts 6
      3.1.5 Time of the best response 8
      3.1.6 Correlation between densitometric 20% and mRECIST 9
4. DISCUSSION 10
5. CONCLUSIONS 11
LIST OF ABBREVIATIONS 11

1. INTRODUCTION

This short report aims to optimize the still non validated Choi's et al [49] radiological densitometric response assessment method in Hepato-Cellular Carcinoma (HCC) treated with Trans Arterial Radio Embolization (TARE), through the comparison with the consolidated mRECIST method [50]. The application of the Choi's et al method to HCC treated with sorafenib was proposed by Gavanier et al [51]. Both methods are applied here to assess the response of a single lesion in relationship to absorbed dose predicted with ^{99m}Tc SPECT/CT. The complete definition of a densitometric method capable of ascertaining the oncological patient response is not developed yet, and is beyond the aim of this work. The first problem in the development of a densitometric response assessment method is the definition of the optimal threshold of variation of density (HU) to define a response.

The study summarized in this short report was preliminary to the study presented in the main text. It analysed different patient cohorts.

2. METHODS

2.1. Densitometric method: search for the best density variation threshold

Patients analysed in this short report belong to the two HCC cohorts A and B defined in [52]. They had been treated with ^{90}Y glass microspheres on Thursday of the first week after the reference time (3.75 days decay interval). These two groups differed in planning method. A was treated following the standard manufacturer's indication [53], while B with personalised dosimetry [52]. More important, for group A the mRECIST evaluation was not available [53]. Follow-up evaluations were performed at 1, 3, 6, 9…months with multiple phase CT scan with contrast medium with two scanners, PHILIPS Brilliance (64

slices) and SIEMENS Somaton flash (128 slices), using the same acquisition protocol. Two radiological criteria were applied for single lesion response assessment: the consolidated mRECIST and a variation of the densitometric method by Choi et al [49], proposed for HCC [51] but non validated after TARE. In the latter, radiologist drew a circular ROI in one arterial phase section of tumour and reported the mean HU number. Attention was paid to exclude the outer border where the actinic rim may simulate an increased enhancement.

The variation with respect to the original Choi's et al method was the % density variation threshold necessary to define partial response (PR). Choi et al adopted 15%. In the following we report our extensive investigation regarding the optimal density variation threshold to define a radiological response. Complete response (CR) was defined as the disappearance of lesion in the arterial phase or the absence of enhancement compared with the scan without contrast medium. From this definition, CR lesions according to the densitometric method are a subset or coincide with CR at mRECIST. Partial response (PD) was defined as a HU density reduction larger than the threshold (to be determined), or the reduction of RECIST diameter > 10%. Progression of disease (PD) was defined as in Choi et al as an increase in diameter > 10% without sufficient density reduction. Stable disease (SD) means that criteria for CD, PR or PD are not met. The best response was considered, and the time of best response was reported.

To obtain the optimal densitometric threshold we performed a ROC analysis. For this purpose, a clinical criterion is necessary, capable of defining a really responding lesion.

In order to obtain such a gold standard, as first attempt we reverted the Gavanier's et al method [51]. We stratified patients in true responding and true non-responding with the cut-off on Overall Survival of 16.2 months, the median OS of the studied population. In patients having more than one target lesion, only the lesion with the worst Choi response was considered, assuming that this would have compromised the OS. As limit of this short report, note that we included patients subject to medical actions before or after the first TARE (additional TACE, sorafenib, RF ablation…, resection). Such actions might have influenced their OS. Only transplantation censored patients from the analysis. Table S1 lists such treatments for patients evaluated with the densitometric criterion. Treatment in columns are not exclusive, i.e. some patient received more than one additional treatment to TARE.

Table S1. patients evaluated for response. Treatments additional to first TARE complicate the relationship between response to 1st TARE and overall survival.

No of patients with densitometric evalation	79	RFA	resection	sorafenb	TACE	PEI	CT/genc	TARE	OLT
Treatments before TARE	No.	18	14	8	28	2	1		
	%	23%	18%	10%	35%	3%	1%		
Treatments concomitant with TARE	No.			6					
	%			8%					
Treatments after TARE	No.			21	6		1	7	3
	%			27%	8%		1%	9%	4%
Treatment between 1st TARE and best response	No.			3	3				
	%			4%	4%				

2.2. Search for the best lesion absorbed dose threshold

The search for the best HU threshold gave uncertain results, probably for the above reported reported methodological limits. We therefore tested four possible values: 15%, 20%, 36.5%, 50%. For each of these values, the optimal absorbed dose threshold for response was investigated with a second ROC analysis. Depending on the aim, a scientist may set the optimal lesion dose threshold where the Youden index (sensitivity + specificity) is maximal, or to have the highest positive predictive value.

3. RESULTS

3.1. The best response criterion and the optimal dose threshold

3.1.1. Lesion volumes and ^{99m}Tc MAA predicted absorbed doses

Table S2 reports the volume and the absorbed doses of all the studied lesions. 79 patients of cohort B were analysed. Among 89 lesions chosen as the best to be measured 79/89 (89%) were measurable with mRECIST, 86/89 (97%) with density. 76 were evaluated with both methods.

Table S2. distributions of lesion volumes and MAA predicted absorbed doses.

Kind of lesions		All	nodular
Cohort		B	B
No.of lesions		89	45
Minimmum vohme	[cm^3]	3	3
25%Percentile		37	13
Median volume		106	56
75%Percentile		231	259
Maxinun vohme		1663	1663
Minimum absorbed dose	[Gy]	75	75
25%Percentile		240	203
Median absorbed dose		346	310
75%Percentile		504	432
Maximum absorbed dose		1247	1021

3.1.2. The best density variation threshold with the densitometric method from stratification on the median OS

Following the assumption that true responders and non-responders might be separated by the OS cut-off of 16.2 m, the plot shown in figure S1 was obtained. The two groups have statistically significant difference of median HU variation at non-parametric Mann Whitney test (25.5% versus 32%, p=0.039). However, the separation between the two classes is bad, and the AUC under ROC curve is poor, 0.62 with 95% C.I [0.51 to 0.72]. The Youden factor (= sensitivity + specificity), was considered as function of the variation of density threshold value (figure S2). The maximal Youden value, i.e. the best HU threshold, was apparently at 36.5%. However, the following sections will show that this threshold value is not reliable, probably because it was determined starting from OS of patients who received too many and heterogeneous additional treatments. We tested other HU threshold values.

Figure S1. lesion density variations in CT arterial phase (basal - best response value). Patient were stratified according to OS cut-off of 16.2 months, the median value of the population.

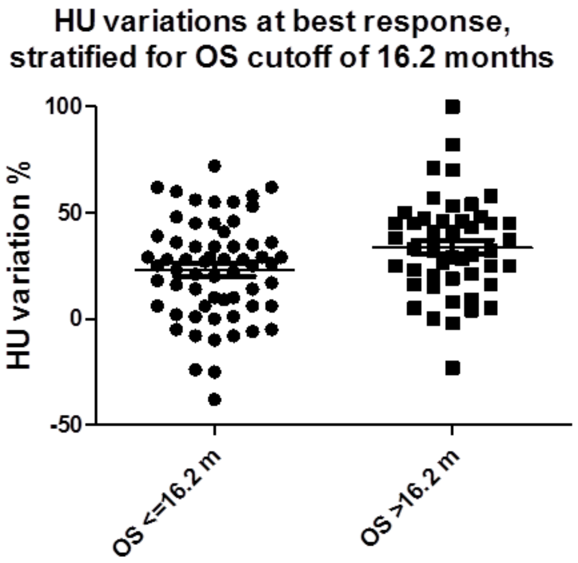

Figure S2. Youden factor = Sensitivity + Specificity, as a function of the density variation threshold. The maximum Youden factor was at an HU reduction of 36.5 %.

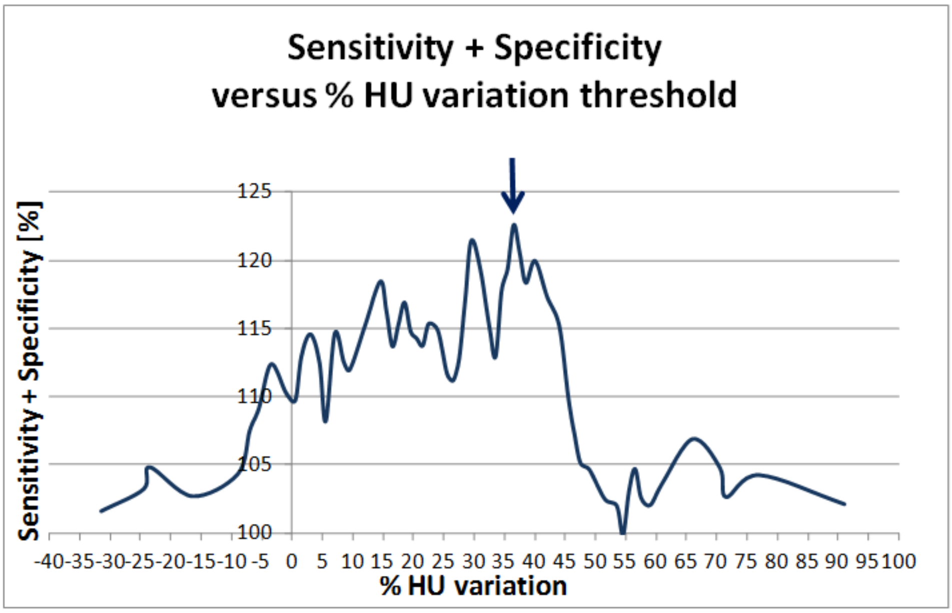

3.1.3. Further search of the best dose threshold in densitometric criterion compared with mRECIST

Four possible threshold values were considered: 15% (the original value by Choi et al [49]), 20%, 36.5% (obtained from OS stratification) and 50%. Their results were compared with the results obtained with mRECIST (figure S3). The 50% threshold, used in our previous paper [53], was excluded from figure S3, demanding an excessive density reduction, and giving a response rate too low compared to the other methods (Table S3).

Figure S3 contains the essence of the problem of HCC lesion prognosis after TARE based on dosimetry. No response criteria showed good separation between responding and non-responding lesions in terms of absorbed dose, except the tails at high absorbed dose of the responding lesions.

Table S3 compares the four considered HU thresholds in terms of response rate, median dose difference between responding and non-responding lesions, AUC under ROC curve, and optimal lesion absorbed dose threshold. Youden index in table S3 is maximal for specificity approaching to 100% , i.e. for an absorbed dose threshold set just above the maximal dose of non-responding lesions. The only reasonable dose threshold value seems just above the highest dose of non-responding lesions (500 Gy for HU threshold equal to 15%, 20%, 36.5%). This choice gives a negligible false responding rate in dosimetric prediction. The positive/negative predictive power obtained with the indicated absorbed dose threshold are also reported. These values of predictive power are valid under the reported prevalence of responding lesions.

As expected, the response rate with the densitometric method monotonically decreased for an increasing density threshold. It was 82%, 74%, 41%, 23% for density response threshold of 15%, 20%, 36.5%, 50% respectively. The corresponding positive predictive value is 100% with 15% and 20% density threshold. mRECIST optimal dose threshold is at 380 Gy (maximal sensitivity + specificity)with 88% positive predictive value.

The best performances in table 3 were obtained with the 20% HU threshold: lowest Mann-Withney p-value of 0.008, highest AUC value of 0.69 and best agreement with mRECIST in terms of response rate (74% versus 73%).

The p-value of AUC for HU threshold value at 15% and at 50% were not statistically significant (p-value=0.14 and 0.60 respectively). This means that you cannot reject the hypothesis that AUC=0.5. In other words, nor the original threshold by Choi's et al (15%) [49] nor our previous value (50%) [53] can give reasonable responses other than random choices, like flipping a coin. Using such either of such threshold, lesion absorbed dose would not able to discriminate between responding and non-responding lesions. With the latter value the median of responding lesion was lower than the median of non-responding (333 Gy vs 361 Gy). Response thresholds at 15% and 50% must therefore be discarded.

Figure S3. dosimetric comparison of responding and non-responding lesions of cohort B according to 3 different thresholds for the densitometric criteria and for mRECIST.

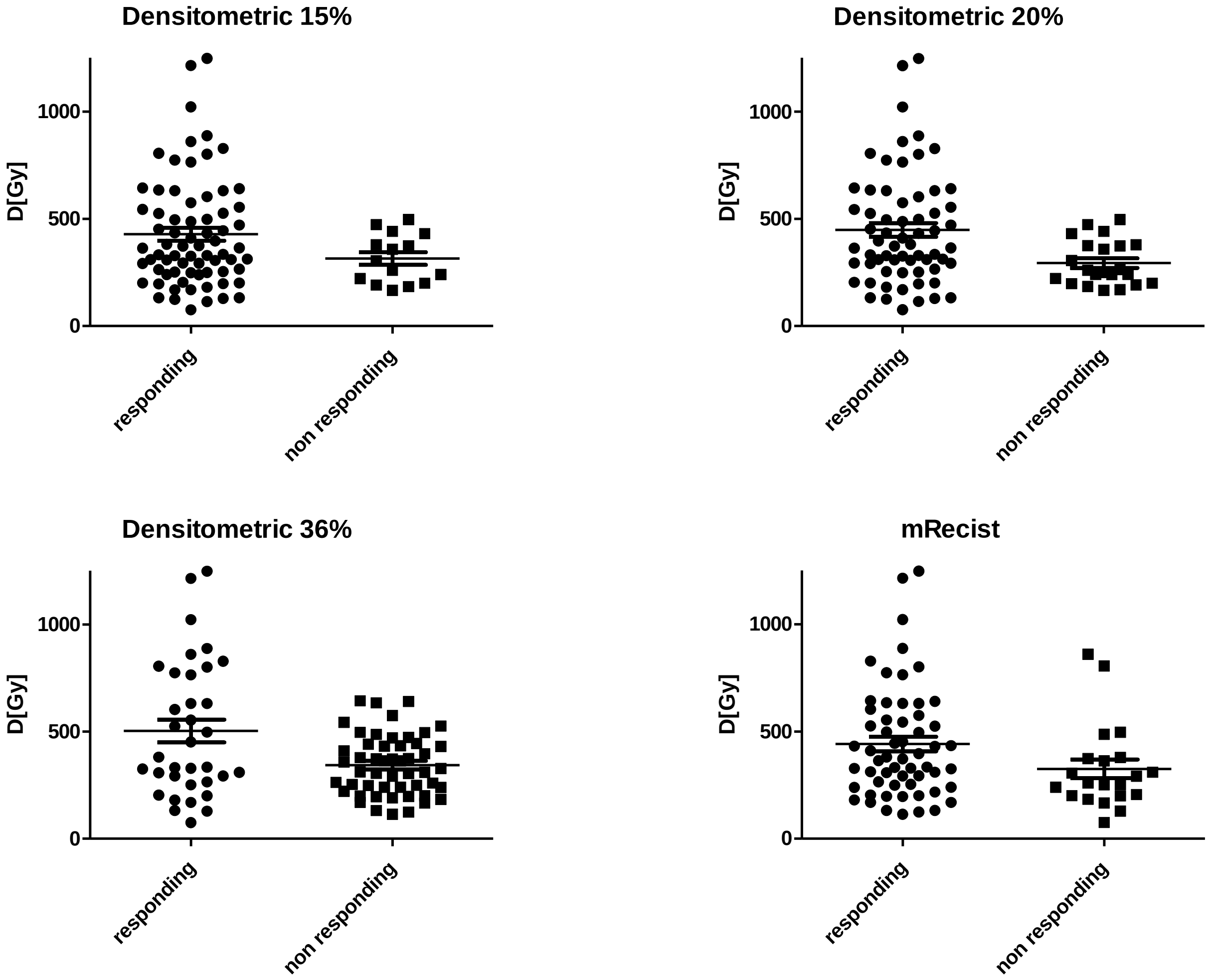

3.1.4. Analysis of merged cohorts

In order to increase the number of observations, we merged data of both cohorts and analysed them with the most promising threshold at 20% for the densitometric criterion (last column in table 3). Figure S4 shows the two classes (responding = CR + PR, non-responding = SD + PD). AUC is 0.76 with 95% C.I of [0.68 to 0.83] (merged cohorts) versus 0.65 [0.52 0.78] for mRECIST (cohort B only). Fixed the densitometric criterion with 20% threshold, the best absorbed dose threshold could be chosen at the maximal Youden index, at 380 Gy, with a positive predictive value of 91%. Alternatively it could be set to reach the positive predictive power of 100% (absence of false positive, no false prediction of responding lesion), i.e. just above the maximal lesion dose of the non-responding group, at 500 Gy (red line in figure S4). The methodological limit is that this threshold value is determined only by the highest single absorbed dose among the non-responding lesions.

Table S3. comparison of response criteria. Densitometric method with different HU threshold and mRECIST result are shown. Nodular, and infiltrative were included. The absorbed dose threshold for response corresponds to the maximum Youden index (sensitivity + specificity). The last column merged data of both cohorts, analysed with the most promising threshold at 20% HU reduction. Note the improvement of the densitometric method with respect to mRECIST in terms of p- and AUC values.

	HU variation in arterial phase				mRECIST: max diameter variation in arterial phase	HU variation in arterial phase
Threshold for response	15%	20%	36.5%	50%	30%	20%
Cohort	B	B	B	B	B	A&B
No. of evaluate lesions	86	86	86	86	79	146
No. of responding lesions	71	64	35	20	58	106
Response rate (CR+PR)(%)	82%	74%	41%	23%	73%	73%
Median dose of responding lesions[Gy]	363	377	381	333	377	436
Median dose of non responding lesions[Gy]	305	255	328	361	260	246
Mann Withney t-test p value	0.14	**0.008**	**0.04**	0.60	**0.04**	<0.0001
Area under ROC curve	0.62	0.69	0.63	0.54	0.65	0.76
Std. Error	0.069	0.058	0.05	0.083	0.069	0.04
95% confidence interval	0.49 to 0.76	0.58 to 0.80	0.50 to 0.76	0.38 to 0.70	0.52 to 0.78	0.68 to 0.83
P value	0.14	**0.008**	**0.04**	0.59	**0.04**	<0.0001
Maximal Youden index (sens. + spec.): optimal dose threshold to discriminate response	500 Gy	500 Gy	500 Gy	700 Gy	380 Gy	380 Gy
Sensitivity at maximal Youden index	30%	33%	43%	30%	50%	58%
Specificity at maximal Youden index	100%	100%	88%	94%	81%	85%
Positive predictive value at max Youden index	100%	100%	71%	60%	88%	91%
Negative predictive value at max Youden index	23%	34%	69%	82%	37%	43%
Prevalence (responding/total)	83%	74%	41%	23%	73%	73%
Maximal dose of non responding lesions						500 Gy

Sensitivity at 500 Gy dose threshold	38%
Specificity at 500 Gy dose threshold	100%
Positive predictive value at 500Gy dose threshold	100%
Negative predictive value at 500Gy dose threshold	38%

Figure S4. absorbed dose of 146 responding and non-responding lesions according to densitometric response criterion with 20% density variation threshold. Setting the dose threshold at 500 Gy we had 100% positive predictive value in this data sample.

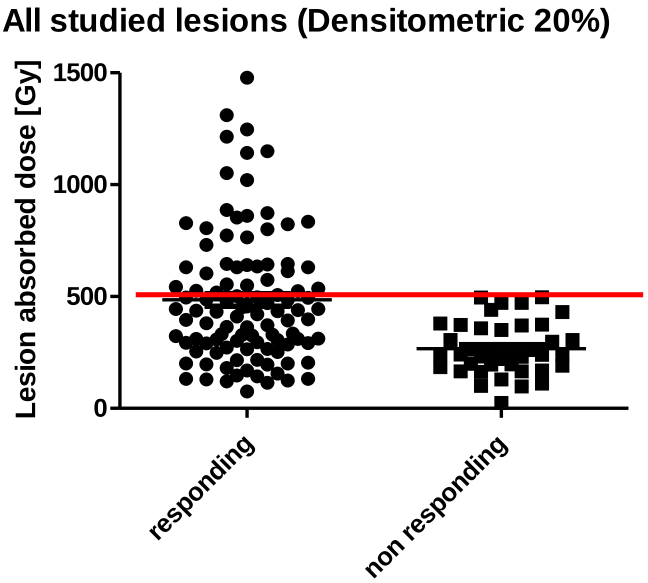

3.1.5. Time of the best response

A tiny difference between median time of response between the two methods is evidenced by the paired t-test: 5.4 months (densitometric 20%) versus 5.2 months (p=0.02). Figure S5 plots times of the best responses as points of coordinates X (mRECIST time of the best response) and Y (densitometric time of the best response). While a series of lesions showed the same time of best response with the two methods (points aligned on the bisector), 14 are scattered above the bisector, indicating longer time to reach the best response with the densitometric method, and 7 vice versa.

Figure S5. the time of the best response as determined with the two criteria.

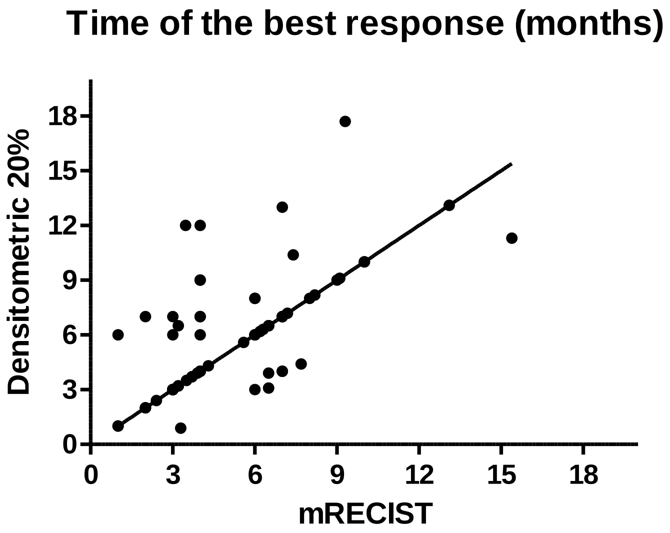

3.1.6. Correlation between densitometric 20% and mRECIST

76 lesions evaluated with both criteria are plotted as points in figure S6 with coordinates X=mRECIST % variation, and Y=density % variation at best response. Actually, points are rather scattered on the plot. At the rightmost position (X=100%) 24 lesions showed no enhancement, and therefore were classified as CR for both methods. After stratification of lesions in responding and non-responding, the association between the two methods is significant at Fisher's exact test (p=0.01). Response rates are reported in table S4.

Figure S6. correlation between densitometric and mRECIST reductions.

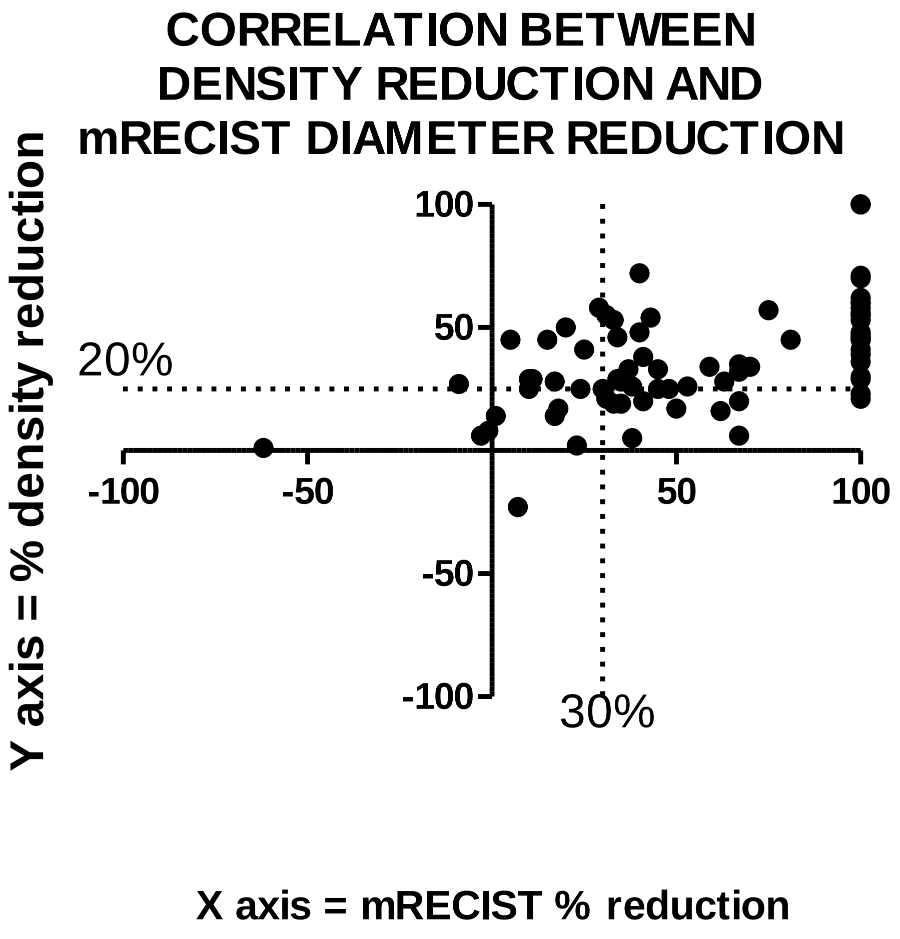

Table S4. Correlation between the two response assessment methods. The association between the two methods in categorizing responding versus non-responding lesions is significant at Fisher's exact test (p=0.01).

	Densitometric 20%		mRECIST	
No. of studied lesions	86		79	
	NO.	%	NO.	%
CR	26	34%	27	34%
PR	36	47%	31	39%
SD	13	17%	20	25%
PD	1	1%	1	1%
OR(CR+PR)	62	72%	58	73%
DCR(CR+PR+SD)	75	87%	78	99%

4. DISCUSSION

Data developed in the present study demonstrated that the 50% threshold used in our previous dosimetric paper [53] was too high. We then tried to derive the best threshold from the stratification of patients on their median overall survival (16.2 months) and we obtained a 36.5% threshold. This value was then abandoned, since it provided lower response rate than mRECIST, and above all a worse separation between the classes with respect to the 20% threshold (table S2). Failure of the analysis based on OS probably derived from the fact that patients were too heterogeneous both in their basal conditions

(PVT, NON PVT, nodular, infiltrative and mixed lesions), and in the sequence of therapies additional to TARE (table S1).

Among the tested thresholds of the densitometric criterion (table S3), the value of 20% seems the most promising. It shows the highest significance of difference between median dose of responding vs non-responding lesions (377 Gy vs 255 Gy, p=0.008), the maximal AUC = 0.69 with 95% C.I. [0.58, 0.80] and response rates similar to mRECIST method (table S3).

Moreover, in HCC TARE, it offers the advantage of being applicable in a higher percentage number of lesions than mRECIST (97 % versus 89%). It is probably less operator dependent, since in HCC TARE the identification of maximal diameter with enhancement sometime is tough with mRECIST.

The time to reach the best response has a broad distribution with its maximum at 3 months, but with a long tail prolonging up to 12 months. This long response time should be considered in trials since too many authors limit their evaluations to 3 months. The densitometric method requires more frequently a longer follow-up interval to reach the best response than mRECIST (figure S6).

Once the preferred radiological response assessment method is chosen, the question is how to set the lesion dose threshold for response. In this work we focussed on 100% positive predictive value (PPV), which means to predict a sure response. However, any method based on 100% sensitivity (Garin et al), 100% specificity, 100% PPV, is too sensible to individual lesion dose value. In our case, 500 Gy gave 100% PPV and specificity, being the highest dose of non-responding lesion. This holds with this lesion sample. In another patient population you could find a non-responding lesion with more than 500 Gy. Note that this dose threshold appears so high since it corresponds to 100% Tumour Control Probability (TCP_{100}). Usually the lower TCP_{50} is assumed as efficacy threshold.

5. CONCLUSIONS

This pilot study investigated an embrio of a new response criterion based on the densitometric reduction ≥ 20% as response threshold for a single lesion analysis. This is a promising alternative to mRECIST in ^{90}Y TARE of HCC that needs to be largely refined but deserves to be furtherly investigated and clinically validated.

CR	Disappearance of lesion, OR 100% HU lesion density reduction, OR absence of enhancement
PR	HU density reduction ≥ 20%, OR reduction of the maximal diameter > 10%
SD	Does not meet the criteria for CR, PR, or PD
PD	An increase in tumor size ≥ 10% and does not meet criteria of PR by tumour density (HU) on CT

For both methods, the time to best response has maximal frequency at 3 months, but it prolongs up to 12 months and beyond.

Using the densitometric 20% method, an efficacy threshold at the maximal dose of non-responding lesions (500 Gy) gave positive predictive value = 100%. This high value correspond to TCP_{100}, while usually the lower TCP_{50} is considered as efficacy threshold.

Dose threshold fixed as above are too dependent on the particular lesion. Planning should rather consider TCP curves.

LIST OF ABBREVIATIONS: D = lesion mean absorbed dose [Gy], DCR = Disease Control Rate, FU = follow up, Gy = Gray, unit of absorbed dose, HU = Hounsfield Units, NPV = Negative Predictive Value, OR = Objective Response, OS = overall survival, PPV = Positive Predictive Value
